# Transcriptomic and Targeted Carotenoid Metabolomic Dissection of Pod Color Difference Between Wild Type and an EMS-Induced Stable *pcm* Mutant in Snap Bean (*Phaseolus vulgaris* L.)

**DOI:** 10.3390/ijms27188380

**Published:** 2026-09-20

**Authors:** Qiuhui Mu, Hongning Wu, Guojun Feng, Xiaoxu Yang, Dajun Liu, Zhishan Yan, Taifeng Zhang, Chang Liu

**Affiliations:** College of Advanced Agriculture and Ecological Environment, Heilongjiang University, Harbin 150000, China; mqh0928@126.com (Q.M.); whn18834291848@126.com (H.W.); feng998@126.com (G.F.); 2017028@hlju.edu.cn (X.Y.); 2016003@hlju.edu.cn (D.L.); 1994028@hlju.edu.cn (Z.Y.); 2024056@hlju.edu.cn (T.Z.)

**Keywords:** snap bean, deep-yellow pod color mutant, carotenoid, transcriptome sequencing, metabolome sequencing

## Abstract

Pod color is an essential economic trait of snap bean (*Phaseolus vulgaris* L.), and golden-yellow pods are more commercially popular than light-yellow varieties. Exploring the molecular regulatory mechanism underlying deep-yellow pod formation is essential for snap bean quality improvement. In this study, the wild-type cultivar Jinguan and the EMS-derived deep-yellow pod mutant *pcm* were used as materials. Integrative analyses of photosynthetic pigment quantification, genetic analysis, transcriptome sequencing, and targeted carotenoid metabolomics were performed to identify key genes and metabolic pathways governing pod color variation. Genetic analysis revealed that the deep-yellow pod trait of the *pcm* is stably inherited and controlled by a nuclear recessive gene. Transcriptome screening identified three differentially expressed key genes, PSY (*Phvul.006G024100g*), CCD4 (*Phvul.002G120600g*), and CYP707A (*Phvul.002G122200g*), whose expression patterns were strongly correlated with carotenoid accumulation. Metabolomic results demonstrated that the *pcm* presented reduced upstream β-carotene content, while the synthesis and accumulation of downstream chromogenic carotenoids, including zeaxanthin and capsorubin, were significantly enhanced, resulting in elevated total carotenoid content. Collectively, the differential expression of these genes remodels carotenoid metabolism and ultimately contributes to the deep-yellow pod phenotype. This study explores the regulatory mechanism of carotenoid metabolism in pod color formation, providing valuable theoretical support and genetic resources for quality breeding of snap beans.

## 1. Introduction

The main edible part of the snap bean (*Phaseolus vulgaris* L.) is the tender pod. The color of the pod significantly affects the vegetable’s marketability. The color of the plant’s fruits, leaves, and flowers depends on the relative concentrations of chlorophyll, carotenoids, anthocyanins, and other pigments. Leaf chlorophyll strongly influences plant leaf color. Chlorophyll in fruits affects nutritional value and flavor. Promoting chloroplast development boosts photosynthesis and increases sugar accumulation. These changes support plant growth and development [1,2]. Anthocyanins are a class of flavonoid compounds; their types and concentrations are key factors in the coloration of the fruits, flowers, and vascular tissues of different plants [3]. Carotenoids are widely distributed in nature, including in higher plants, algae, microorganisms, and molluscan shells [4]. In addition to playing a role in photosynthesis, carotenoids also impart color to flowers and fruits; yellow, orange, and red hues all stem from high levels of carotenoid accumulation. These pigments not only enhance the visual appeal of plants but also promote seed dispersal, thereby facilitating plant reproduction [5]. In terms of plant growth and development, carotenoids also serve as precursors for the synthesis of important hormones, contribute to the formation of aromatic and flavor compounds in flowers and fruits, and act as signaling molecules involved in the feedback regulation of carotenoid metabolism, amongst other functions [6].

Carotenoid metabolism primarily takes place in plant plastids and involves both biosynthetic and degradative pathways. To date, significant progress has been made in understanding the molecular mechanisms that regulate carotenoid metabolism; however, current research has primarily focused on the leaves of the model plant *Arabidopsis thaliana* and the fruits of horticultural crops such as tomatoes and citrus. Carotenoid compounds are initially derived from the plastid-localized 2-C-methyl-D-erythritol 4-phosphate (MEP) pathway (distinct from the cytosolic mevalonate, MVA pathway), where various precursors are utilized to produce geranylgeranyl diphosphate (GGPP). GGPP is the direct precursor for carotenoid synthesis and also serves as a precursor for isoprenoids such as gibberellins (GA), chlorophyll, tocopherol, phylloquinone (VK1) and plastoquinone (PQ) [7]; under the action of phytoene synthase (PSY), GGPP condenses to form colorless phytoene, which is further converted into red lycopene under the catalysis of phytoene desaturase (PDS), ζ-carotene desaturase (ZDS), ζ-carotene isomerase (Z-ISO) and carotene isomerase (CRTISO); the carotenoid biosynthetic pathway diverges into two major branches downstream of lycopene. Within the β-branch, β-carotene undergoes successive hydroxylation reactions to yield β-cryptoxanthin and zeaxanthin, which may serve as substrates for the biosynthesis of capsorubin. In the α-branch, α-carotene is subjected to hydroxylation modifications to generate lutein [8]. In the ε-carotenoid branch, α-carotene can be converted into lutein under the action of cytochrome P450-type hydrogenases [9].

For example, in citrus plants (such as Wenzhou mandarin or sweet orange), the high accumulation of carotenoid compounds like β-carotene, β-cryptoxanthin, lutein, and β-citraurin induced by ethylene significantly contributes to the orange or red coloration of the fruit peel [10]; studies on red or yellow tomato fruits indicate that the increased expression levels of the phytoene synthase gene (*PSY*) and phytoene desaturase gene (*PDS*) are among the reasons for the elevated carotenoid content [11]; research shows that white-heading cabbage has significantly lower carotenoid content than orange-heading cabbage, highlighting the crucial role of carotenoids in determining fruit color and leaf pigmentation. However, research into carotenoid metabolism has largely focused on citrus or vegetable crops, whilst studies on the biosynthesis and regulation of carotenoids in cowpeas and other crops remain relatively limited.

The color and shape of the pods are important agronomic traits of snap beans, directly influencing consumer preferences and the market value of the product. Mutants play an indispensable role in elucidating the biosynthesis, regulation, and functional diversity of carotenoids in plants [12]. In snap beans, mutants play a crucial role in elucidating the genetic basis of pod color variation and nutritional quality; in particular, inactivating mutations in key carotenoid synthesis genes such as *PSY*, *PDS*, and *LCYb* lead to metabolic blockages and phenotypic changes, thereby providing direct genetic evidence of gene function [1,13]. To further elucidate the genetic basis of pod traits, Garcia-Fernandez et al. [14] studied 301 legume accessions using both monogenomic and multi-genomic genome-wide association studies (GWAS) to systematically investigate key pod characteristics, including pod color and pod shape. They ultimately identified eight candidate genes involved in pigment synthesis, providing important insights into the genetic regulation of pod color. In mutant research, a study on the purple pod mutant of snap beans showed that the *PV-PUR* mutant gene directly affects the formation of the purple phenotype in bean pods by regulating the biosynthesis and accumulation of anthocyanins. This discovery provides key genetic resources for breeding and molecular marker-assisted selection of purple pod traits in snap bean [15]. In addition, the study of the golden pod bean strain ‘A18-1’ revealed that the formation of the golden pod phenotype is closely associated with a decrease in chlorophyll content. This study not only clarified the phenotypic causes of golden pod color but also provided important references for analyzing the biochemical mechanisms of pod color changes [16].

In summary, previous studies on snap bean pod color have predominantly focused on the metabolic pathways of anthocyanins and chlorophyll, whereas systematic investigations into carotenoid-mediated deep-yellow pod formation are still insufficient. The key regulatory genes and precise metabolic networks linking carotenoid accumulation to pod phenotypic differentiation remain poorly characterized, which greatly restricts the in-depth understanding of snap bean pod color regulation and quality improvement. In view of this research gap, the wild-type variety Jinguan and its Ethyl methanesulfonate (EMS)-induced deep-yellow pod mutant *pcm* were used as materials in this study, which is conducive to exploring the molecular mechanism of carotenoid-dependent pod color difference. In order to test the above hypothesis, we measured the content of photosynthetic pigment during pod wall development, analyzed its genetic law, and selected the period with the most obvious phenotypic difference for transcriptome and targeted metabolome analysis. Based on the data of physiological phenotype, genetic map, transcriptomics and metabolomics, the molecular mechanism of carotenoid metabolism driving pod color differentiation was systematically clarified. The results enrich the theoretical system of carotenoid regulation in legumes and provide valuable genetic resources and a theoretical basis for breeding high-quality legumes with good pod color and nutritional traits.

## 2. Results

### 2.1. Analysis of Photosynthetic Pigment Content in Two Varieties of Snap Beans

**Figure 1:**
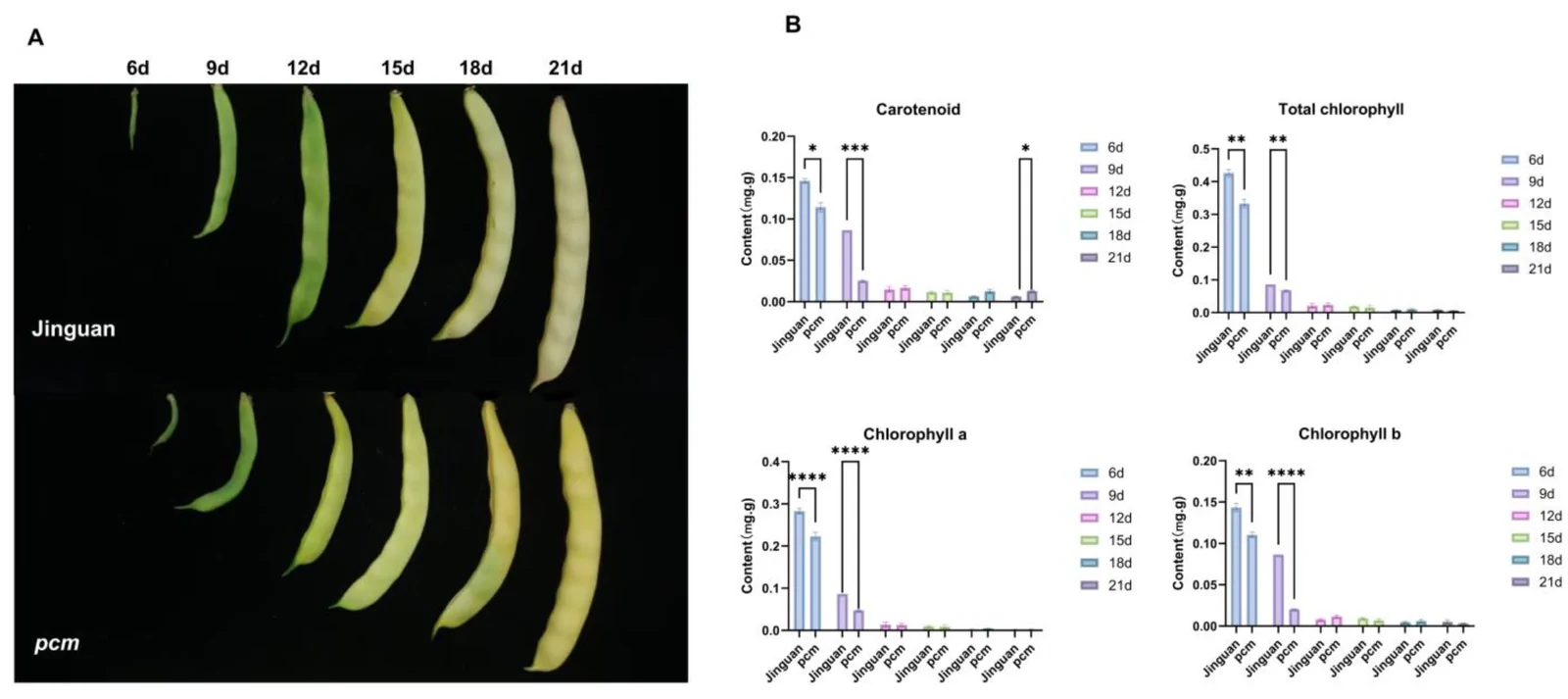
Comparison of parental materials. (**A**) Pod development of Jinguan and *pcm* 6, 9, 12, 15, 18 and 21 days after flowering. (**B**) The pigment content and trend of the pod wall of the two materials on days 6, 9, 12, 15, 18, and 21 after flowering,. Data represent the mean ± standard error (*n* = 3). Asterisks indicate significant differences between genotypes at each time-point (* *p* < 0.05, ** *p* < 0.01, *** *p* < 0.001, **** *p* < 0.0001; Šidák test).

The tested plant materials, Jinguan and mutant *pcm*, are both early-maturing yellow pod dwarf varieties, with flattened round pods and light-yellow pods. Compared with Jinguan, the mutant *pcm* has the characteristic of a deep-yellow pod color. The phenotype of bean pods is shown in Figure 1A. Generally, bean pods remain green for 9 days after flowering and gradually change color from 12 to 15 days after flowering. The color change time varies with growth conditions. Eighteen days after flowering, the pods had turned completely yellow, while the pods of the *pcm* were a more pronounced deep yellow 18–21 days after flowering. Measurements of photosynthetic pigment content in Jinguan and the mutant *pcm* revealed that chlorophyll a (Chl-a), chlorophyll b (Chl-b), total chlorophyll (Chl), and carotenoids all showed a decreasing trend as the pods developed (Figure 1B). Six and nine days after flowering, photosynthetic pigment levels in Jinguan remained higher than those in the mutant *pcm*; however, starting from 12 days after flowering, pigment levels in the mutant *pcm* exceeded those in Jinguan. At 21 days after flowering, the carotenoid content in the mutant *pcm* was significantly higher than that in Jinguan (Table 1, Figure 1B). Although no statistically significant difference in carotenoid content was detected at 21 days, the *pcm* consistently showed deeper yellow pod color at this stage. Full statistical outputs of Šidák multiple-comparison tests are listed in Table S1. Based on these results, it is inferred that the formation of deep-yellow pods is caused by increased carotenoid content.

### 2.2. Inheritance of the Deep-Yellow Pod Mutant

The pod color of the F_1_ generation produced by the reciprocal cross of Jinguan and the mutant *pcm* was yellow, and the F_2_ generation had trait segregation, with a total number of 543 plants, including 399 plants with the normal yellow phenotype and 144 plants with the deep-yellow phenotype. After statistical analysis of the F_2_ population phenotype, it was found that the segregation ratio was 3:1 (χ^2^ = 0.669) (Table 2). The results indicate that the deep-yellow pod mutant trait is controlled by a single recessive nuclear gene, which has been named *pv-pcm*.

### 2.3. Multinomic Analysis Revealed That the Carotenoid Synthesis Pathway Played an Important Role in the Development of Deep-Yellow Pods

#### 2.3.1. Transcriptome-Wide Gene Expression and Differentially Expressed Genes (DEGs) Between Jinguan and *pcm*

**Figure 2:**
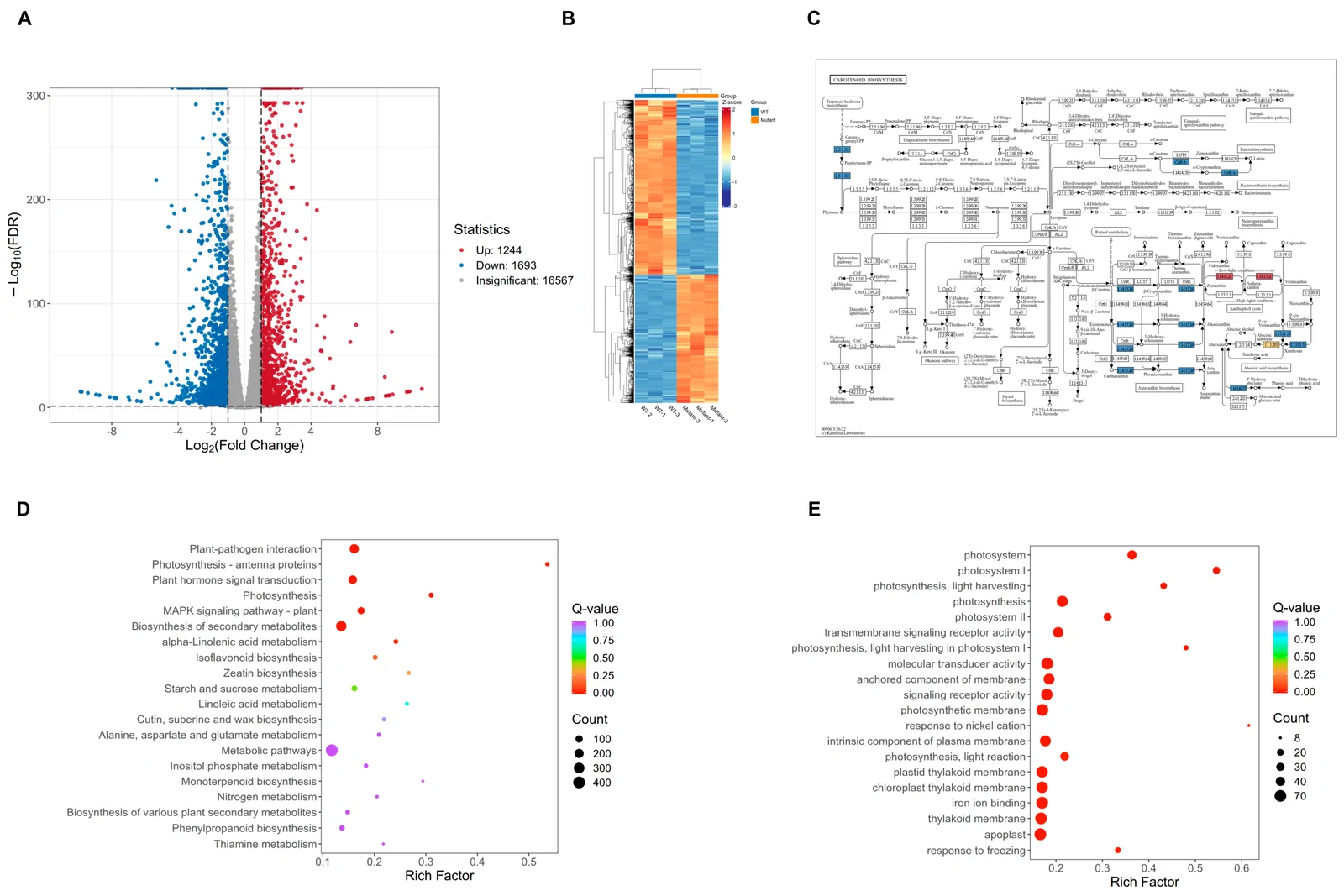
Transcriptomic analysis. (**A**) Volcano plot of DEGs. (**B**) Hierarchical clustering heatmap of differentially expressed genes (DEGs). The heatmap is plotted based on log_2_-transformed FPKM values of DEGs. The color gradient represents normalized gene expression levels; red indicates high expression and blue indicates low expression. Each row represents one DEG, and each column corresponds to an individual biological sample. (**C**) KEGG enrichment pathway diagram for DEGs (ko00906). (**D**) Scatter plot of KEGG enrichment for DEGs. (**E**) Scatter plot of GO enrichment for DEGs.

Sample correlation and principal component analysis (PCA) analyses revealed high intra-group reproducibility in both the experimental and control groups, with significant inter-group differences, indicating that the results are reliable (Figure S1). A total of 275,268,978 clean reads were obtained from the Jinguan and mutant *pcm* samples. The results indicate that read quality is sufficiently high and sequencing quality is satisfactory, making the data suitable for subsequent analysis (Tables S2 and S3). Based on |log_2_Fold Change (FC)| ≥ 1 and false discovery rate (FDR) < 0.05, a total of 2939 genes were annotated. Among these, 1244 (42.33%) were upregulated DEGs, and 1693 (57.60%) were downregulated DEGs (Figure 2A). Furthermore, for DEGs clustered within the same branch, their expression patterns in the two samples—Jinguan and the mutant *pcm*—are very similar (Figure 2B), suggesting they may share similar functions or participate in the same metabolic processes or signaling pathways.

The top 20 Kyoto Encyclopedia of Genes and Genomes (KEGG) pathways with significant enrichment among DEGs in Jinguan and the mutant *pcm* are presented in an enrichment scatter plot (Figure 2D). KEGG pathway analysis indicated that DEGs were primarily associated with plant–pathogen interactions, plant hormone signaling, secondary metabolite biosynthesis, plant mitogen-activated protein kinase (MAPK) signaling pathways, photosynthesis, metabolic pathways, and other metabolic processes. Significant differences in the expression of genes involved in carotenoid biosynthesis were observed across these metabolic pathways. After *p*-value correction (*p* < 0.05), Gene Ontology (GO) enrichment analysis identified a total of 15 GO terms associated with carotenoid metabolism (Figure 2E). The KEGG pathway map shows the DEGs involved in carotenoid biosynthesis and their main pathways. Here, DEGs are mostly found in key steps and later branches, such as the pathway starting with octahydrolycopene, the lutein cycle, and the abscisic acid pathway. This shows groups of related changes in key molecules during carotenoid metabolism and provides pathway-level clues to understanding how carotenoids accumulate in mutants (Figure 2C).

**Figure 3:**
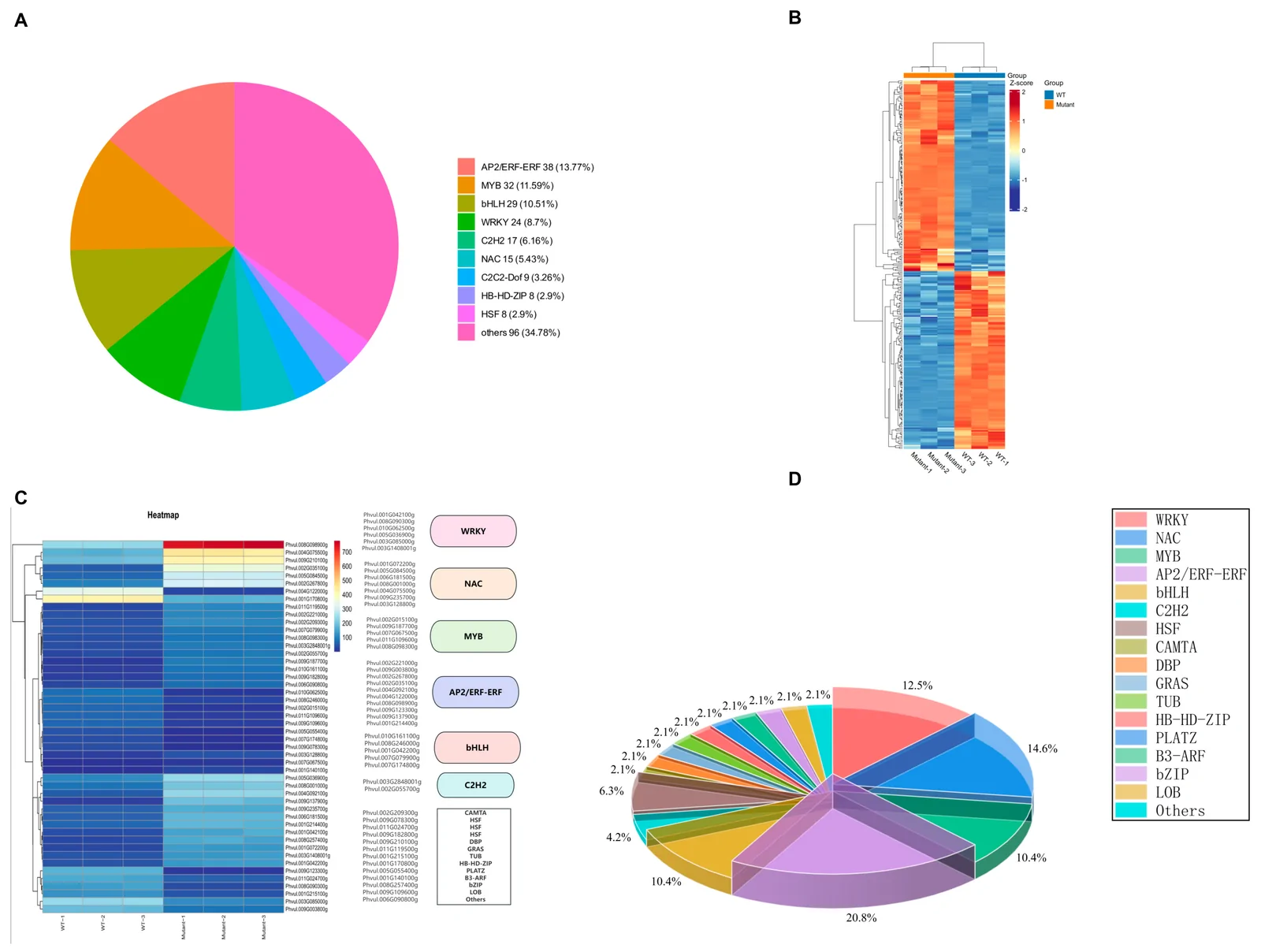
Analysis of differentially expressed transcription factors. (**A**) Pie chart showing the distribution of differentially expressed transcription factor gene families. (**B**) Heatmap of differentially expressed transcription factor clusters. (**C**) Expression heatmap of key candidate transcription factors from representative TF families. (**D**) Family composition proportion of key candidate transcription factors.

Transcription factors are core components that regulate the expression of carotenoid synthesis genes, playing a crucial role in this process. Various transcription factors have been identified that specifically bind to the promoters of structural genes and regulate carotenoid content by activating or inhibiting gene expression. Based on the differential analysis results of each comparison group, detailed annotation information of DEGs was extracted (Table S4, Figure 3C), identifying a total of 58 C3H-type zinc-finger transcription factor (C3H) genes, 168 MYB-domain transcription factor (MYB) genes, 154 basic helix-loop-helix transcription factor (bHLH) genes, 155 APETALA2/ethylene-responsive factor (AP2/ERF-ERF) genes, 125 C2H2-type zinc-finger transcription factor (C2H2) genes, and 77 basic region-leucine zipper (bZIP) genes (Figure 3A).

Transcription factor analysis revealed (Figure 3C,D) that in the snap bean pod color mutant *pcm*, core regulatory transcription factor families involved in carotenoid biosynthesis, such as MYB and bHLH, were significantly downregulated, whereas the AP2/ERF-ERF family was significantly upregulated. In the deep-yellow pod mutant of the snap bean, AP2/ERF-ERF transcription factors were significantly upregulated. By positively regulating the upstream pathways of carotenoid biosynthesis and inhibiting their degradation, they promote carotenoid accumulation, which is directly associated with the darkened-pod phenotype and increased carotenoid content. It is hypothesized that this gene activates key genes involved in carotenoid biosynthesis and inhibits their degradation through a similar mechanism, ultimately leading to increased carotenoid content and darker pod color, a regulatory pattern that is highly conserved across the aforementioned species.

**Figure 4:**
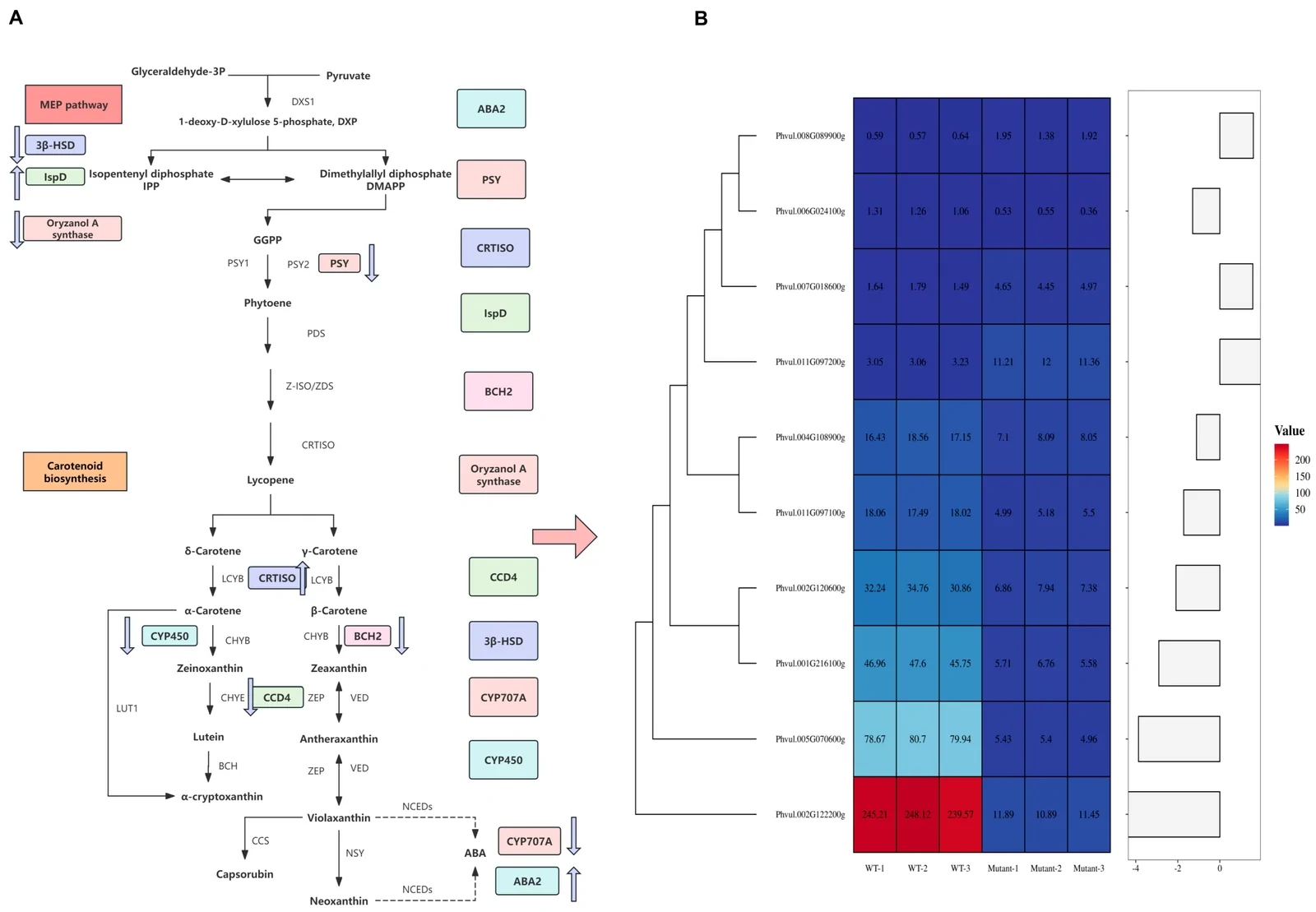
Analysis of carotenoid metabolic pathways and the expression patterns of key DEGs. (**A**) Schematic diagram of carotenoid biosynthesis in *Phaseolus vulgaris*, showing the core pathways of the upstream MEP pathway and downstream carotenoid synthesis. The catalytic steps and product flow of key enzymes/genes in the pathway are labeled; the direction of the arrows indicates the trend in gene expression in *pcm*s (↓: significantly downregulated; ↑: significantly upregulated). (**B**) Heatmap of key DEGs in the carotenoid pathway in wild-type (WT1–3) and *pcm*s (Mutant-1–Mutant-3). Heatmap of key carotenoid-metabolism-related DEGs based on log_2_-transformed FPKM values. Red indicates high expression; blue indicates low expression across WT and *pcm* samples.

To reveal the molecular basis of the mutant *pcm* phenotype formation, genes related to carotenoid metabolism were analyzed based on the selection criteria of |log_2_FC| ≥ 1 and FDR < 0.05, resulting in the identification of 10 DEGs directly involved in carotenoid metabolism (Table S4). The pathway schematic diagram (Figure 4A) illustrates the core genes involved in carotenoid metabolism and their regulatory network. The heatmap (Figure 4B) further demonstrates significant differences in the expression of several key genes involved in carotenoid metabolism between the Jinguan and the mutant *pcm*. These results indicate that the overall expression pattern of carotenoid metabolism-related genes in the mutant *pcm* has changed significantly, providing important clues for further elucidating the molecular regulatory mechanism underlying this mutant’s phenotype.

**Figure 5:**
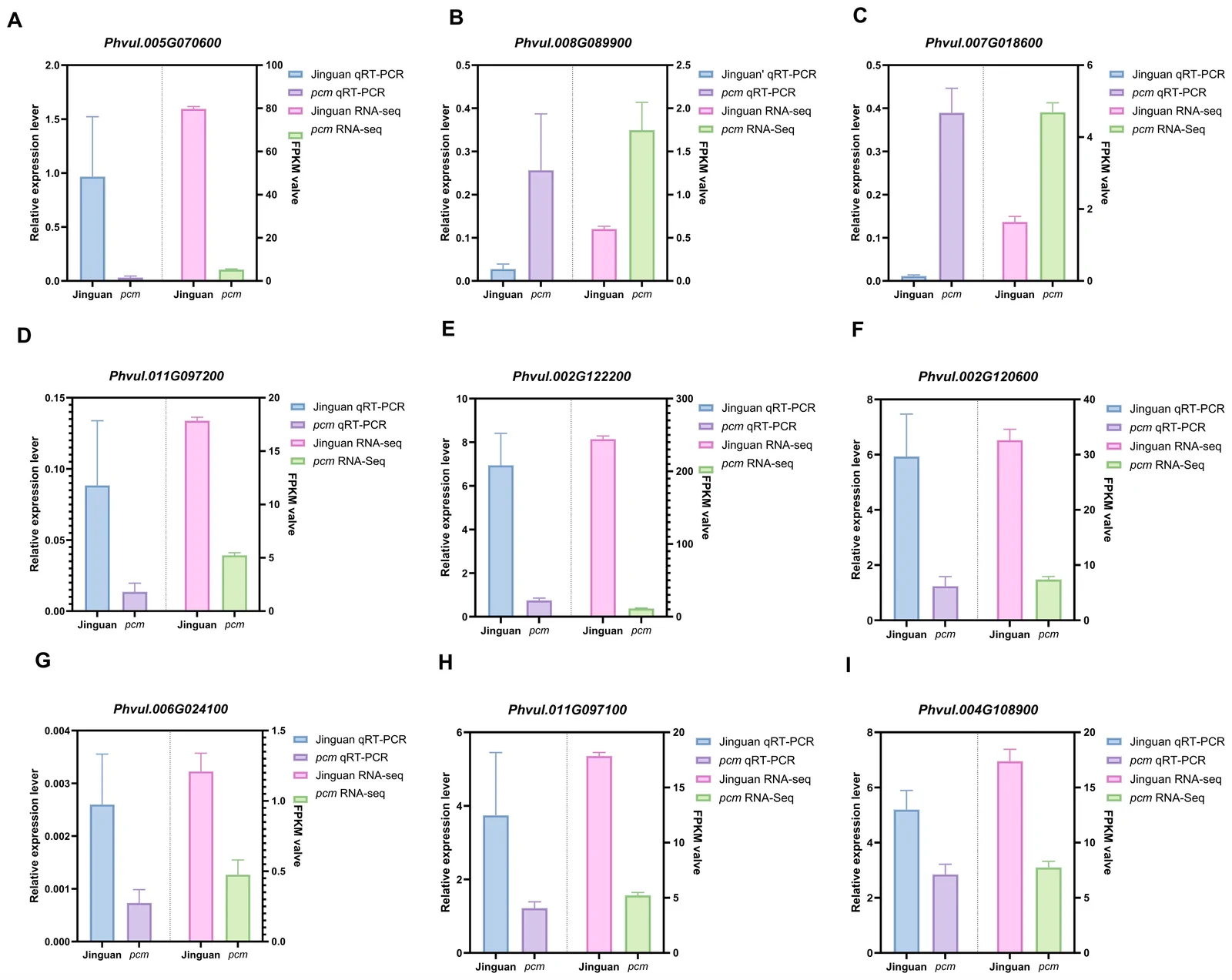
Expressions of DEGs between Jinguan and *pcm*. (**A**–**I**) Comparison of gene expression levels measured by qRT-PCR and RNA-seq for nine selected genes. (**A**) *Phvul.005G070600g*; (**B**) *Phvul.008G089900g*; (**C**) *Phvul.007G018600g*; (**D**) Phvul.011G097200g; (**E**) Phvul.002G122200g; (**F**) *Phvul.002G120600g*; (**G**) *Phvul.006G024100*; (**H**) *Phvul.011G097100g*; (**I**) *Phvul.004G108900g*. The blue and purple bar charts on the left show the relative expression levels obtained from qRT-PCR analysis. The pink and green bar charts on the right show the FPKM values obtained from RNA-seq analysis.

To verify the reliability of the transcriptomic expression data, we selected nine DEGs associated with carotenoid metabolism and validated their expression levels by quantitative real-time polymerase chain reaction (qRT-PCR). The expression trends observed in the qRT-PCR results were consistent with those from RNA sequencing (RNA-seq) (Figure 5), further confirming the accuracy and reliability of the transcriptomic data. In particular, the genes *Phvul.002G122200g*, *Phvul.002G120600g*, and *Phvul.004G108900g* encoding abscisic acid 8′-hydroxylase (CYP707A), carotenoid cleavage dioxygenase 4 (CCD4), and beta-carotene 3-hydroxylase 2 (BCH2) have a regulatory relationship with carotenoid synthesis, showing significantly higher relative expression levels in Jinguan compared to *pcm* (Figure 5). Among them, downregulation of *CCD4* reduces carotenoid degradation, while downregulation of *CYP450* and *BCH2* leads to accumulation of beta-carotene. The downregulation of *CYP707A* may increase endogenous abscisic acid (ABA) levels, promoting carotenoid accumulation in a feedback loop. These three genes exhibit expression patterns closely associated with the deep-yellow phenotype of the *pcm* [17]. In contrast, the genes *Phvul.008G089900g* and *Phvul.006G024100g*, which encode the enzymes abscisic aldehyde oxidase (ABA2, involved in abscisic acid biosynthesis) and PSY, involved in carotenoid biosynthesis), show significantly lower relative expression levels in Jinguan compared to *pcm* (Figure 5).

#### 2.3.2. Targeted Metabonomic Analysis of Carotenoids

The heatmap generated from standardized data (Figure S2B) shows significant metabolic differences between the two varieties. The distribution of coefficient of variation (CV) values for quality control samples further confirms the instrument’s stability and the reliability of the data (Figure S2A).

**Figure 6:**
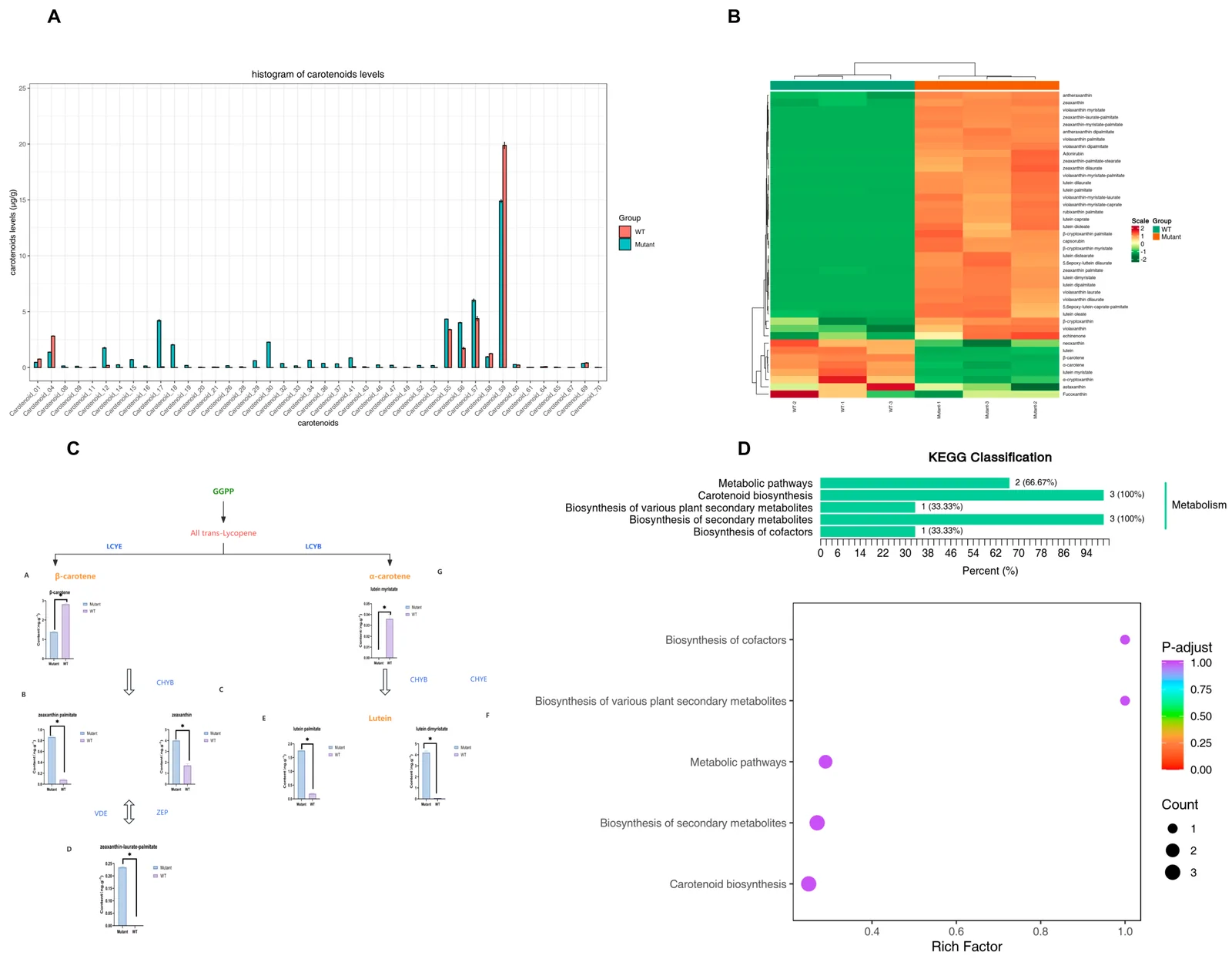
Carotenoid profiling in pods of Jinguan and *pcm* snap bean. (**A**) Histogram of carotenoid content in different varieties of pod-type carrots. (**B**) Carotenoid composition in the pods of various snap bean varieties. ‘WT’ stands for Jinguan; ‘Mutant’ stands for *pcm*. The color scale represents the normalized content of each carotenoid component. (**C**) Main differential metabolite content identified through KEGG pathway screening. Asterisk indicates significant difference between wild-type Jinguan and *pcm* by Student’s *t*-test (* *p* < 0.05). (**D**) KEGG classification and pathway enrichment of DEMs.

Targeted metabolomics analysis identified a total of 42 carotenoid compounds from pods of the Jinguan and mutant *pcm* samples (Figure 6). Differential-accumulated carotenoid metabolites were screened using combined criteria: VIP > 1 from the OPLS-DA model, *p* < 0.05 (*t*-test), and fold-change ≥ 2 or ≤0.5. Using these criteria, 31 differential carotenoid metabolites were obtained (Table S6, Figure 6B). The more abundant differentially expressed metabolites (DEMs) included β-carotene, zeaxanthin palmitate, zeaxanthin-laurate-palmitate, zeaxanthin, lutein palmitate, lutein dimyristate, and lutein myristate (Figure 6C). Among these metabolites, β-carotene and lutein myristate were significantly downregulated in the *pcm*, whereas zeaxanthin, zeaxanthin-laurate-palmitate, zeaxanthin palmitate, lutein palmitate and lutein dimyristate were significantly accumulated.

The annotation results for significantly enriched DEMs were classified and enriched by KEGG pathway type. The results showed that DEMs were mainly annotated to the carotenoid biosynthesis pathway, plant secondary metabolite biosynthesis pathway, metabolic pathways, and cofactor biosynthesis pathways (Figure 6D, Table S7).

### 2.4. Integrated Transcriptomic and Metabolomic Analysis Confirm That Carotenoid Biosynthesis Is the Key Reaction Pathway

**Figure 7:**
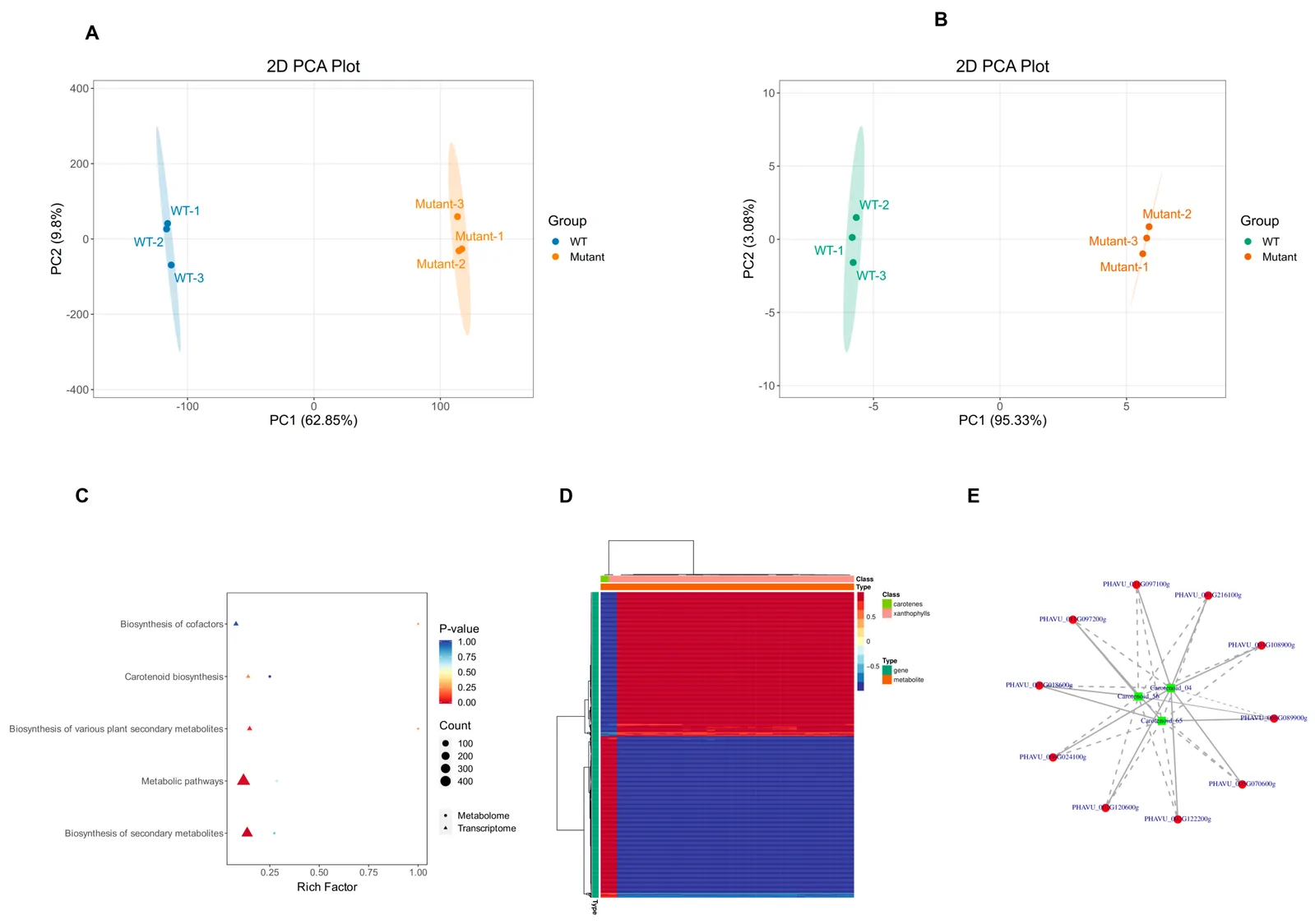
Combined transcriptomic and metabolomic analysis. (**A**) Transcriptome PCA plot. (**B**) Metabolomics PCA plot. (**C**) KEGG enrichment analysis bubble chart. (**D**) Correlation clustering heatmap. Each row in the figure represents a gene, and each column represents a metabolite. Red indicates a positive correlation between the gene and the metabolite, whilst blue indicates a negative correlation. (**E**) Correlation network diagram. ‘WT’ stands for Jinguan; ‘Mutant’ stands for *pcm*.

A joint principal component analysis (PCA) of transcriptome and metabolome data revealed significant separation of ‘WT’ and ‘Mutant’ samples in the principal component space, reflecting synergistic differences at the omics level (Figure 7A,B).

KEGG enrichment analysis was performed on the transcriptome and metabolome data. In cases where the number of enriched pathways exceeds 25, the top 25 pathways can be selected based on *p*-values with transcriptome data as a reference for visualization. The results indicate significant differences between these two omics datasets in the statistical significance and enrichment levels of the enriched pathways. Notably, the differential enrichment of the carotenoid biosynthesis pathway provides key clues for studying carotenoid metabolic regulation (Figure 7C). A correlation clustering heatmap was used to identify gene–metabolite relationships with similar patterns, selecting DEMs and DEGs with r ≥ 0.8 to explore potential associations between genes and metabolites. Correlation clustering heatmap analysis shows that DEGs associated with carotenoid metabolism cluster significantly positively with DEMs, with genes and metabolites forming independent clusters, reflecting a coordinated response between transcriptional regulation and metabolite accumulation within this pathway (Figure 7D).

Significantly correlated DEGs and DEMs were screened using Pearson’s correlation coefficient with the thresholds of |r| > 0.8 and *p* < 0.05. A multi-omics association network was then constructed to visualize the key metabolites involved in the carotenoid metabolic pathway (Carotenoid_04-β-carotene, Carotenoid_56-zeaxanthin, and Carotenoid_65-paprika red pigment) as well as their regulatory genes (*Phvul.001G216100g*, *Phvul.002G122200g*, *Phvul.002G120600g*, *Phvul.004G108900g*, *Phvul.005G070600g*, *Phvul.006G024100g*, *Phvul.007G018600g*, *Phvul.008G089900g*, *Phvul.011G097100g*, *Phvul.011G097200g*) (Figure 7E).

In this study, transcriptome and carotenoid targeted metabonomics analysis identified PSY (*Phvul.006G024100g*), CCD4 (*Phvul.002G120600g*), and CYP707A (*Phvul.002G122200g*). The expression of key genes such as β-carotene, zeaxanthin, capsorubin, etc. thus reshapes the carotenoid metabolic flux, causing differences in key metabolites such as β-carotene, zeaxanthin, and capsorubin and finally mediating the color differentiation between *pcm* and Jinguan pods.

## 3. Discussion

Carotenoids are important secondary metabolites in plants and are involved in various physiological processes, including photoprotection [18], antioxidant activity, and growth and development. The color of the snap bean pod is one of its important commodity traits. With the improvement of modern living standards, people have higher requirements for the edible quality and appearance of snap beans. However, the molecular mechanisms of carotenoid synthesis, accumulation, and regulation in the pod still lack systematic research. This study utilized stable *pcm*s selected via EMS mutagenesis that exhibit distinct color differentiation during development compared to the wild type. Results from photosynthetic pigment assays at different developmental stages indicate that the deep-yellow pod phenotype of the *pcm* is caused by an increase in total carotenoid content and changes in specific components within the carotenoid group and is not closely related to chlorophyll a and b. This is consistent with the findings of CONESA et al. [19], whose results indicate that the ripening of lemon peel is caused by the degradation of chlorophyll and an increase in carotenoids, particularly an increase in β-cryptoxanthin. Meanwhile, research by ZHANG et al. [20] on cabbage mutants suggests that their yellow-green coloration is associated with a decrease in chlorophyll and carotenoid content. By establishing an F_2_ population to analyze genetic patterns, we confirmed that the deep-yellow pod trait in the *pcm* is controlled by a single recessive nuclear gene, thereby clarifying the genetic characteristics of this trait. A study has identified a large number of key differentially expressed genes involved in carotenoid biosynthesis, confirming that the yellowing and coloration of green bean pods during the late stages of pod maturation are synergistically regulated by chlorophyll degradation and the stepwise accumulation of carotenoids; among these, differences in carotenoid metabolism are the key factor determining the formation of the yellow color in the pods [21].

Through a combined transcriptomic and carotenoid-targeted metabolomic analysis, this study revealed the gene–metabolite synergistic regulatory network underlying the color differentiation of pods between the wild-type Jinguan and the *pcm*. The core determinant of the depth of yellow color in snap bean pods is the dynamic balance between carotenoid synthesis and degradation, and changes in the expression of DEGs—PSY (*Phvul.006G024100g*), CCD4 (*Phvul.002G120600g*), and CYP707A (*Phvul.002G122200g*)—were significantly correlated with the accumulation of carotenoid components in the pods. A comprehensive pathway analysis revealed that the DEGs and DEMs identified in this study are concentrated in the core steps of carotenoid biosynthesis and downstream branch pathways. These primarily include the carotenoid biosynthesis pathway starting with octahydrolycopene, the lutein cycle, and the abscisic acid biosynthesis pathway, which are key functional pathways regulating pigment accumulation in snap bean pods. Previous studies have shown that *PSY1* improves tomato fruit quality by altering the distribution between carotenoid and flavonoid pathways [22]; research on wheat conducted by HE indicates that *PSY* genes regulate the content of yellow pigments in grains, thereby influencing the quality of wheat products [23]; and in Anji White Tea, PSY—which is involved in carotenoid biosynthesis—shows a significant positive correlation with carotenoid content [24]. In addition, *PSY* can significantly influence the direction and efficiency of downstream derivative synthesis by regulating the overall progression of the carotenoid metabolic pathway [25,26,27]. CCD4 is a key enzyme in the carotenoid degradation pathway. Previous studies have identified several structural genes involved in this pathway, including *CsCCD1s*, *CsCCD4*, *CsCCD7s*, *CsCCD8s*, *CsD27s*, and *CsNCEDs*. These genes use carotenoids as substrates and cleave them to produce various derivatives. In this study, we found that *Phvul.002G120600g* (*CCD4*) is highly expressed in the wild-type Jinguan variety. We hypothesize that, in the pod wall tissues of the two varieties, *CCD4* cleaves carotenoids to produce β-carotene derivatives, thereby leading to differences in pod carotenoid content and phenotype. Transcriptomic profiling further revealed that *CCD1*, *CCD7* and *CCD8* exhibited either negligible transcript levels or non-significant expression differences between wild-type and *pcm* pods in our materials. According to previous functional characterization, plant *CCD1* primarily generates volatile apocarotenoid aroma compounds rather than directly determining tissue pigment intensity [6,28]. By contrast, *CCD7* and *CCD8* function sequentially in strigolactone biosynthesis and are not regarded as primary contributors to fruit or pod color formation [29,30]. Thus, they were not considered key candidates underlying pod color divergence, though their functional roles in other snap bean tissues or earlier developmental stages cannot be excluded. *CYP707A* is a key gene family within the plant cytochrome P450 superfamily and one of the central regulators of the ABA metabolic pathway. ABA is an important plant hormone derived from the carotenoid metabolic pathway and is a key derivative of carotenoids. Some studies in chili peppers have reported that altered *CYP707A* expression can change endogenous ABA levels [31]. Whether altered expression of this gene could further modulate carotenoid accumulation through ABA-dependent homeostatic regulation remains speculative in the present study, given that endogenous ABA levels were not quantified in snap bean pod tissues. Physiological, genetic, and molecular changes that occur during fruit ripening lead to alterations in primary and secondary metabolism [32]. During development, the degradation of chlorophyll is accompanied by the biosynthesis of carotenoids and anthocyanins, which determine the color changes in the fruit as it ripens [33]. Targeted metabolomic analysis of carotenoids revealed significant differences in the levels of various individual carotenoid compounds between the *pcm* and the wild type, indicating that the difference in pod color between the two is not only reflected in total carotenoid content but is also accompanied by a reshuffling of the proportions of individual carotenoid components. High *PSY* expression in the wild type activates the carotenoid biosynthetic pathway. The pronounced in vivo upregulation of *CCD4* and *CYP707A* accelerates carotenoid catabolism and may trigger alterations in ABA-associated signaling; however, this interpretation is inferred exclusively from transcriptomic evidence and remains to be corroborated by hormonal measurements. This transcriptional expression pattern shifts carotenoid metabolism in wild-type pods toward catabolism, ultimately resulting in a large accumulation of β-carotene, while the levels of zeaxanthin and the putative capsorubin signal are significantly lower, leading to a pale yellow pod phenotype. In contrast, in the *pcm*, the expression of all three key genes was significantly downregulated, which drastically inhibited carotenoid catabolism. Potential downstream effects of altered ABA-related signaling on pigment accumulation cannot be confirmed in the absence of ABA quantification. Although the expression of *PSY*, the rate-limiting gene for carotenoid biosynthesis, was slightly reduced in the mutants, and upstream biosynthetic capacity was marginally weakened, the substantial decline in degradation efficiency dominated the metabolic rebalancing. This significantly promoted the accumulation of downstream branch metabolites, ultimately leading to a significant upregulation of zeaxanthin and capsanthin, while β-carotene levels decreased, leading the mutant pods to accumulate large amounts of specific carotenoid metabolites and exhibit a deeper yellow phenotype. Previous studies have confirmed that the yellowing of maturing snap bean pods is the result of the joint regulation of chlorophyll degradation and carotenoid accumulation; building on this, the present study further refines the specific molecular regulatory module governing carotenoid metabolism in legume pods.

**Figure 8:**
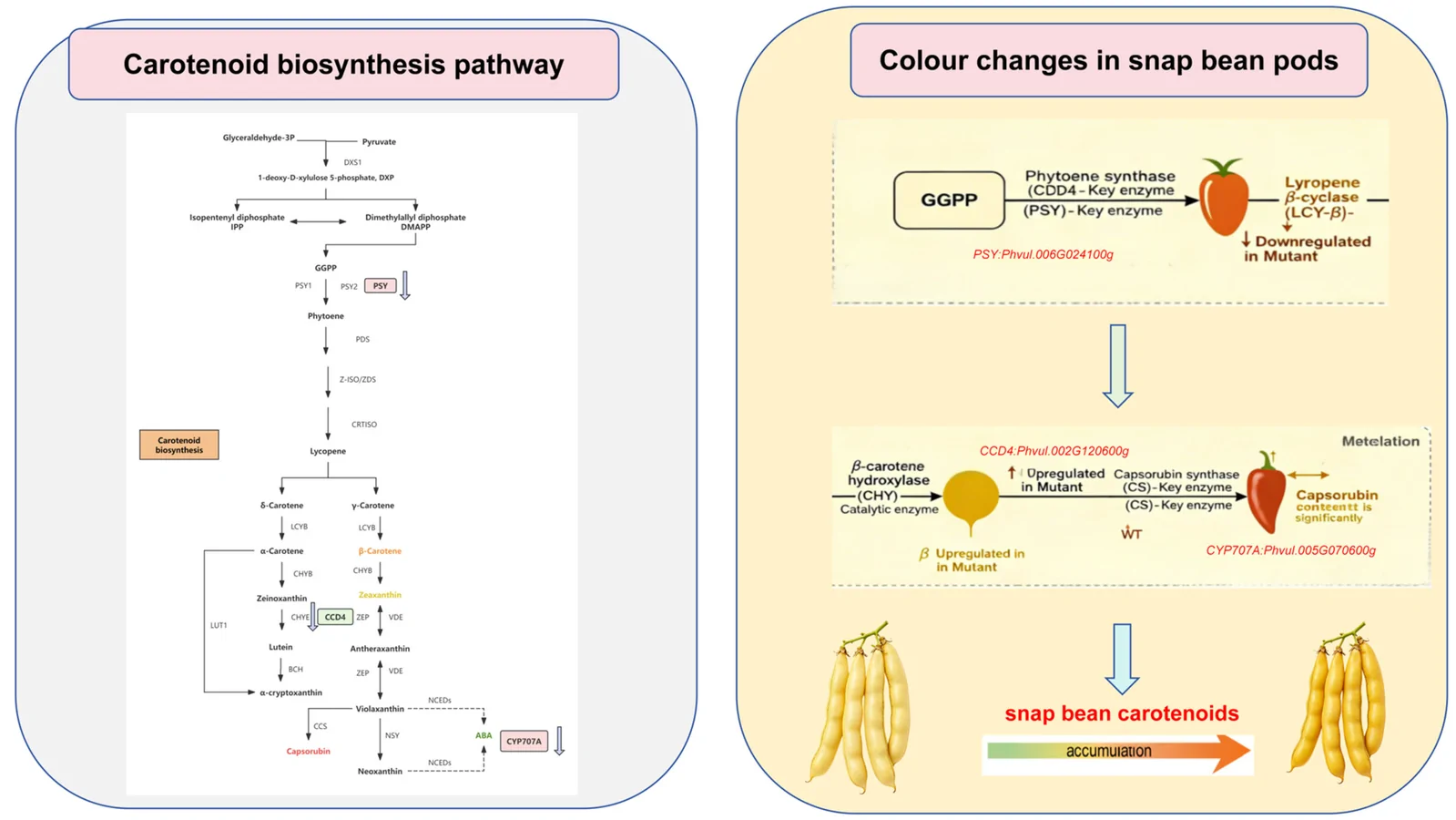
Schematic diagram of carotenoid metabolism and pod color variation mechanism in snap bean pods. PSY: phytoene synthase, CYP707A: ABA 8′-hydroxylase, CCD4: carotenoid-cleaving dioxygenase 4, LCYB: lycopene beta cyclase, CHYB: beta-carotene hydroxylase gene, CHYE: ε-carotene hydroxylase, ZEP: zeaxanthin epoxidase, VDE: violaxanthin de-epoxidase, BCH: β-carotene hydroxylase, CCS: capsorubin synthase, NSY: neoxanthin synthase, NCEDs: 9-cis-epoxycarotenoid dioxygenase.

This study reveals that the expression of the synthetic rate-limiting gene *PSY*, the key degradation gene *CCD4*, and the ABA-metabolism-related gene *CYP707A* are closely correlated with carotenoid metabolism in snap beans. Based on transcriptomic and metabolomic data, these three genes may participate in regulating metabolic fluxes in the core carotenoid synthesis, lutein cycle, and ABA synthesis pathways and may contribute to metabolite differences between mutant and wild-type pods (Figure 8). Further genetic validation is required to confirm their exact regulatory mechanism.

## 4. Materials and Methods

### 4.1. Experimental Materials

The *pcm* (pod color mutant) was derived from EMS mutagenesis of the snap bean cultivar Jinguan. All plant materials were cultivated in the greenhouse at the Horticultural Experimental Base, Hulan Campus of Heilongjiang University. Wild-type Jinguan seeds were treated with 0.9% EMS solution for 6 h to construct the mutant library, generating 3000 M_1_ plants and a self-pollinated M_2_ segregating population of 8452 individuals. No phenotypic screening was performed at the M_1_ generation because most phenotypic alterations in M_1_ seedlings result from non-heritable physiological damage; heritable mutant screening was therefore carried out in the M_2_ population. A total of 373 mutant individuals covering seven trait categories were identified, with an overall population mutation rate of 5.32%. This value is slightly higher than those reported in previous snap bean EMS mutant libraries, possibly owing to differences in cultivar genetic background and mutagenesis conditions. The candidate *pcm* exhibiting a distinct deep-yellow pod phenotype was initially screened in M_2_ and further purified by successive selfing up to the M_4_ generation to obtain genetically stable lines. Previous studies have been consistent with the typical characteristics of EMS mutagenesis, which mainly induces single-point mutations throughout the genome, and linked multi-trait variations are often observed in mutant populations [34,35].

### 4.2. Measurement of Photosynthetic Pigment Content

Pods from the two varieties (wild-type Jinguan and mutant *pcm*) with uniform maturity and free of disease symptoms were sampled at 6, 9, 12, 15, 18, and 21 days after flowering. The contents of photosynthetic pigments, including total chlorophyll, chlorophyll a, chlorophyll b, and carotenoids, in the pod wall of snap beans were quantified following the protocol described by Lichtenthaler et al. [36]; three biological replicates were arranged for each treatment.

### 4.3. Analysis of Genetic Patterns

The mutant *pcm* was individually crossed with the wild type ‘WT’ to construct F_1_, and the F_1_ and F_2_ populations were developed. χ^2^ tests were used to identify deviations from the expected 3:1 segregation ratios in the F_2_ populations. A total of 543 pod plants exhibiting the target phenotype were selected from the F_2_ mapping population. Young pods from these plants were then chosen as experimental material. During sampling, samples were rigorously screened to ensure clear differentiation of pod color, and sampling was performed precisely.

### 4.4. RNA-Seq Analysis

Clear phenotypic differences in pod color were observed between the two materials at 21 days after anthesis. Pod wall tissues were then sampled at this developmental stage for total RNA extraction. Total RNA was isolated from frozen pod wall tissues using the RNAprep Pure Plant Total RNA Extraction Kit (TIANGEN Biotech, Beijing, China, Cat. No. DP432) according to the manufacturer’s instructions. Genomic DNA contamination was removed via on-column DNase I digestion. RNA quality was evaluated using the Qubit 4.0 fluorometer (Thermo Fisher Scientific, Waltham, MA, USA) for concentration measurement and the Qsep400 bioanalyzer (Bioptic, New Taipei City, Taiwan, China) for RNA integrity assessment. Qualified RNA samples were sent to Wuhan Maiwei Metabolomics Technology Co., Ltd. (Wuhan, China) for library construction, transcriptome sequencing on the DNBSEQ platform and subsequent bioinformatics analysis. In this study, the *Phaseolus vulgaris* v2.1 genome (https://genome.jgi.doe.gov/portal/, accessed on 14 September 2026) was adopted as the reference genome. Clean reads were obtained by filtering raw sequencing data with fastp software (version 0.23.2) and mapped to the reference genome using HISAT2 (version 2.2.1) GitHub [37]. Gene-level read counts were generated by featureCounts (Subread package, version 2.0.3) [38]. The raw unnormalized count matrix was used as input for DESeq2 v1.42; R v4.4.1to identify DEGs with thresholds of FDR < 0.05 and |log_2_FC| ≥ 1. FPKM values were calculated subsequently and only used for visualization of gene expression patterns in heatmaps. The wild-type cultivar Jinguan at 21 days after flowering was defined as ‘WT’, while its pod color mutant was designated as ‘Mutant’.

KEGG pathway enrichment analysis of the DEGs was performed with pathways treated as the statistical unit, and the hypergeometric test was used to screen significantly enriched pathways. Meanwhile, GO term enrichment analysis was performed to identify functionally enriched GO terms associated with these DEGs.

Heatmaps for gene expression and metabolite profiles were generated by Wuhan Maiwei Metabolomics Technology Co., Ltd. using R software (version 4.2.1) with the pheatmap package (version 1.0.12) from cran [39]. Gene expression values were log_2_-transformed before visualization. Row-wise hierarchical clustering was performed based on Euclidean distance.

### 4.5. Targeted Metabolome Analysis of Carotenoids and Integrated Transcriptome–Metabolome Correlation Analysis

A targeted metabolomic analysis of carotenoids in snap bean pod wall tissue was conducted. We weighed 50 mg of freeze-dried ground pod wall and added 0.5 mL of an n-hexane–acetone–ethanol mixed extraction solution (1:1:1, *v*/*v*/*v*) containing 0.01% (g/mL) BHT as an antioxidant. The mixture was vortexed at room temperature for 20 min, then centrifuged at 4 °C and 12,000 r/min for 5 min. The supernatant was collected, the extraction of the pellet was repeated once under the same conditions, and the two supernatants were combined. The combined extract was concentrated and redissolved in 150 μL of dichloromethane. After filtration through a 0.22 μm nylon organic-phase filter membrane, it was transferred to a brown sample vial for LC–MS/MS analysis. The entire extraction process was conducted in the dark to prevent carotenoid degradation. Chromatographic separation was performed using an ExionLC™ AD ultra-high-performance liquid chromatography system with a YMC C30 silica reverse-phase column (3 μm, 100 mm × 2.0 mm i.d.; YMC Co., Ltd., Kyoto, Japan); mobile phase A consisted of methanol–acetonitrile (1:3, *v*/*v*), with 0.01% BHT and 0.1% formic acid added; mobile phase B consisted of methyl tert-butyl ether with 0.01% BHT added; and the gradient elution program was set as follows: 0 min, 100% A; 3 min, 100% A; 5 min, A:B = 30:70; 9 min, A:B = 5:95; 10 min, 100% B; 11 min, 100% B; flow rate 0.8 mL/min; column temperature 28 °C; injection volume 2 μL. Mass spectrometry detection was performed on a QTRAP® 6500+ tandem mass spectrometry platform (SCIEX, Framingham, MA, USA).The APCI atmospheric pressure chemical ionization source was set to positive ion mode with a source temperature of 350 °C and curtain gas (CUR) at 25 psi. Metabolite signals were acquired using timed multiple reaction monitoring (MRM), with declustering potential (DP) and collision energy (CE) optimized separately for each metabolite ion pair. Metabolite identification was achieved by matching the retention times and secondary fragment mass spectra obtained from the experiments with the internal MWDB database at Maiwei. Among these, 14 metabolites were further confirmed using commercial standards, while the remaining carotenoid esters were annotated based on secondary mass spectrometry information from the literature sources in the MWDB database. No external public spectral libraries were used in this study; quantification was performed using the external standard calibration curve method, the isotopically labeled internal standard [^13^C_10_]-β-carotene (catalog number IR-23004-10 mg, Isoreag, Shanghai, China) used for auxiliary correction. Data acquisition was performed using SCIEX Analyst 1.6.3 software, and chromatographic peak integration and quantitative calculations were performed using MultiQuant 3.0.3 software. For targeted metabolomic analysis of carotenoids, multiple reaction monitoring–liquid chromatography–tandem mass spectrometry (MRM–LC–MS/MS) was used to perform direct quantification of pod wall tissue extracts. Supplementary Table S8 records all detailed information for the 42 detectable carotenoids, including MRM ion pair parameters, retention times, calibration data, and the basis for metabolite identification; functional annotation of the identified carotenoid metabolites was performed based on the KEGG database. The screening threshold for DEMs was set at |log_2_FC| ≥ 1 and *FDR* < 0.01, followed by KEGG pathway enrichment analysis of the selected DEMs.

The Pearson correlation coefficient was used to assess the relationship between transcriptomic and metabolomic data. Gene–metabolite pairs with a correlation coefficient R > 0.8 and *p* ≤ 0.05 were retained for subsequent analysis.

### 4.6. qRT-PCR Analysis

Total RNA was extracted from pods 21 days after anthesis from wild type ‘WT’ and *pcm*s using Trizol reagent ((Invitrogen, Carlsbad, CA, USA)). Candidate genes and carotenoid biosynthesis-related genes were selected for qRT-PCR verification. In the RNA-Seq analysis, ten genes related to carotenoid biosynthesis were selected, and cDNA from the wild-type and mutant *pcm* pod walls was used as the qRT-PCR template.

Primer 6.0 software was used to design specific primers, and actin was used as an internal reference. Before selecting the final reference genes, the expression stability of five candidate reference genes *(Skip16*, *Action11*, *Action*, *Ukn1* and *Ukn2*) was evaluated across WT and *pcm* pod wall samples using geNorm [40], which ranks candidates by the average pairwise variation measure M and determines the optimal number of reference genes via the pairwise variation V~n/(n + 1)~. All five candidates showed M < 1.0, with *Skip16* and *Ukn2* being the two most stable (Figures S3–S5). The pairwise variation V~2/3~ = 0.136 was below the recommended cutoff of 0.15, indicating that two reference genes were sufficient. Accordingly, the geometric mean of *Skip16* and *Ukn2* was used as the normalization factor. The first-strand cDNA was synthesized using the Hiscript II Q RT SuperMix for qPCR Kit (Vazyme, Nanjing, China). qRT-PCR amplification was performed on an ABI viia7 real-time PCR system (Thermo Fisher Scientific, Waltham, MA, USA) using 2 × SYBR^®^ Green qPCR mix (Biov, Beijing, China) and following the manufacturer’s operating procedures. The specificity of primers was verified by melting curve analysis. Three biological and technical repetitions were performed. Relative gene expression was quantified using the 2^−^^ΔΔCT^ method [41], with normalization to the geometric mean of *Skip16* and *Ukn2*. See Table S9 for details of DEGs and qRT-PCR primer sequences.

### 4.7. Statistical Analysis

The data were organized using Microsoft Excel 2019, and analysis and graphing were performed using GraphPad Prism and IBM SPSS Statistics 27. The omics analysis figures were provided by Wuhan Maiwei Metabolomics Technology Co., Ltd. (Wuhan, China).

## 5. Conclusions

In summary, this study measured photosynthetic pigment content and performed transcriptomic and targeted metabolomic sequencing on Jinguan and mutant *pcm* pods. The deep-yellow pod phenotype of the mutant showed a significant correlation with total carotenoid content. First, transcriptome analysis showed differential expression of several genes involved in the carotenoid metabolic pathway, including PSY (*Phvul.006G024100g*), CCD4 (*Phvul.002G120600g*), and CYP707A (*Phvul.002G122200g*). The differential expression of a series of functional genes in the carotenoid metabolic pathway ultimately leads to the difference in pod color phenotype between wild-type and mutant strains by regulating the synthesis and accumulation mode of metabolites. In addition, 31 kinds of carotene compounds were identified in Jinguan varieties and their mutants using targeted metabonomic sequencing analysis. Among the 31 compounds, the study showed that the deep-yellow pod was caused by the decrease in upstream β-carotene level and the increase in downstream colored carotenoid (zeaxanthin and capsorubin) synthesis and accumulation, which together led to the increase in total carotenoid content. This study provides valuable data for the biosynthesis of carotenoids in pods, provides a new idea for the study of the biosynthesis mechanism of carotenoids in pods, and provides a reference for the breeding of new varieties of pods with good color quality and high nutritional content.

## Figures and Tables

**Table 1:** Photosynthetic pigment content (mg/g) and its relative ratio of commercial pods (21 d).

Material	Chl a Content	Chl b Content	Total Chl Content	Car Content
Jinguan	0.001911 ± 0.000564	0.003256 ± 0.00049	0.005587 ± 0.001001	0.00661 ± 0.000319 *
*pcm*	0.002322 ± 0.000961	0.005189 ± 0.002129	0.0071 ± 0.002693	0.012618 ± 0.002087

*: significant difference, *p* < 0.05.

**Table 2:** Genetic analysis of the yellow pod phenotype in the hybrid progeny of Jinguan and the mutant *pcm.*

	Total	Wild-Type Plants	Mutant Plants	Segregation Ratio	Chi-Square (χ^2^)
P_1_ (Jinguan)	50	50			
P_2_ (*pcm*)	50		50		
F_1_ (P_1_ × P_2_)	43	43			
F_1_ (P_2_ × P_1_)	55	55			
F_2_	543	399	144	2.77:1	0.669

Chi-square test at the significance level (*p* = 0.05).

## Data Availability

The datasets generated and analyzed during the current study are available in the NCBI repository, https://www.ncbi.nlm.nih.gov/. Specifically: the protein sequence analyzed in this study is available from NCBI RefSeq under accession number XP_007155040.1, and the raw transcriptomic data generated in this study are available in the NCBI Sequence Read Archive (SRA) under BioProject accession number PRJNA1477073.

## Supplementary Materials

The following supporting information can be downloaded at https://www.mdpi.com/article/10.3390/ijms27188380/s1.

## Abbreviations

2-C-methyl-D-erythritol 4-phosphate (MEP); geranylgeranyl diphosphate (GGPP); gibberellins (GA); chlorophyll (Chlorophyll); phylloquinone (VK1); plastoquinone (PQ); phytoene synthase (PSY); phytoene desaturase (PDS), ζ-carotene dehydrogenase (ZDS), ζ-carotene isomerase (Z-ISO); carotene isomerase (CRTISO); Genome-Wide Association Study (GWAS); Ethyl methanesulfonate (EMS); chlorophyll a (Chl-a); chlorophyll b (Chl-b); total chlorophyll (Chl); differentially expressed genes (DEGs); false discovery rate (FDR); Kyoto Encyclopedia of Genes and Genomes (KEGG); mitogen-activated protein kinase (MAPK); Gene Ontology (GO); C3H-type zinc-finger transcription factor (C3H); MYB-domain transcription factor (MYB); basic helix-loop-helix transcription factor (bHLH); APETALA2/ethylene-responsive factor (AP2/ERF-ERF); C2H2-type zinc-finger transcription factor (C2H2); basic region-leucine zipper (bZIP); quantitative real-time polymerase chain reaction (qRT-PCR); RNA sequencing (RNA-seq); abscisic acid 8′-hydroxylase (CYP707A); carotenoid cleavage dioxygenase 4 (CCD4); beta-carotene 3-hydroxylase 2 (BCH2); abscisic acid (ABA); coefficient of variation (CV); differentially expressed metabolites (DEMs); principal component analysis (PCA); lycopene beta cyclase (LCYB); beta-carotene hydroxylase gene (CHYB); ε-carotene hydroxylase (CHYE); zeaxanthin epoxidase (ZEP); violaxanthin de-epoxidase (VED); β-carotene hydroxylase (BCH); capsorubin synthase (CCS); neoxanthin synthase (NSY); 9-cis-epoxycarotenoid dioxygenase (NCEDs); multiple reaction monitoring (MRM); declustering potential (DP); collision energy (CE); multiple reaction monitoring-liquid chromatography-tandem mass spectrometry (MRM-LC-MS/MS).
