# Supplementary material for: Transcriptomic and Targeted Carotenoid Metabolomic Dissection of Pod Color Difference Between Wild Type and an EMS-Induced Stable pcm Mutant in Snap Bean (Phaseolus vulgaris L.)
Source: Int J Mol Sci. 2026 Sep 20;27(18):8380. doi: 10.3390/ijms27188380 (PMC13607725; doi:10.3390/ijms27188380)
Supplement: Supplementary file 1 [file ijms-27-08380-s001.zip › ijms-4525909-supplementary.pdf]

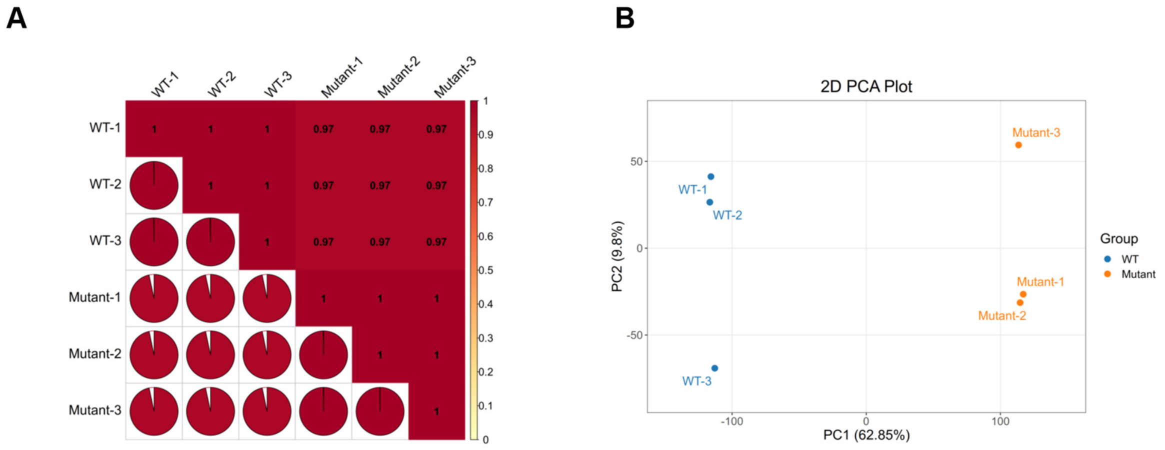

Figure S1. Transcriptomic data analysis. (A) Analysis of sample correlation. The x- and y-axes represent different samples, whilst the color variations in the pie chart indicate the Pearson correlation coefficients between gene expression levels for each pair of samples; the redder the color, the stronger the positive correlation, and the yellower the color, the weaker the correlation. (B) QC sample correlation analysis.

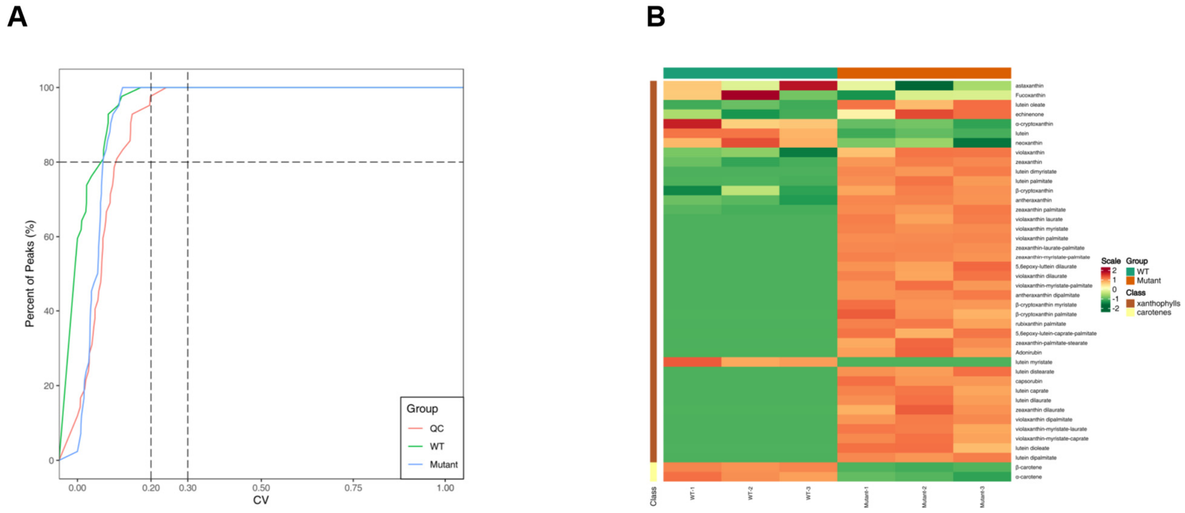

Figure S2. Metabolomics data analysis. (A) Distribution of CV values for QC samples; (B) Clustering heatmap analysis of DEMs for snap bean. The horizontal axis shows the sample names, the vertical axis shows metabolite information, and the different colors represent the values obtained after standardization for different concentrations (red indicates high concentration, green indicates low concentration). 'Group' refers to the grouping, and 'Class' refers to the classification of the substance.

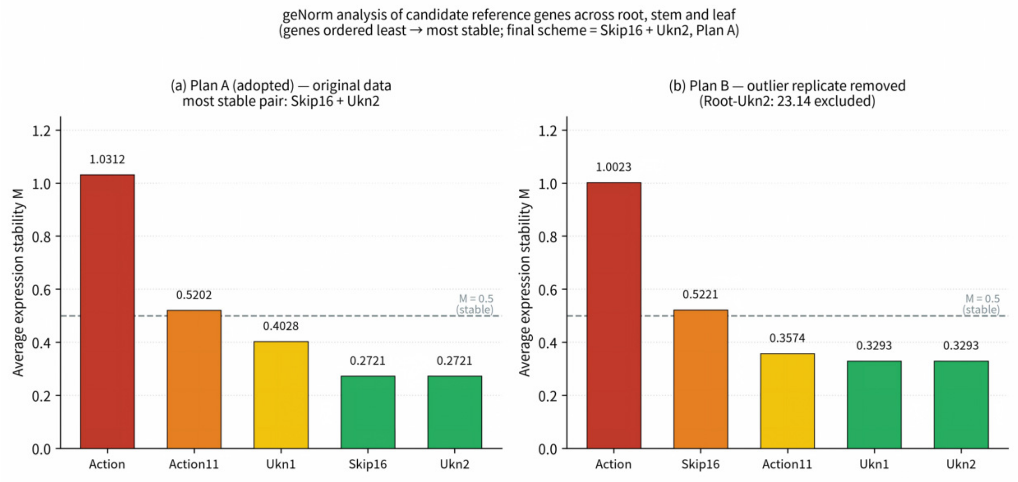

Figure S3. geNorm analysis of candidate reference genes. The average expression stability value (M) was calculated by stepwise elimination of the least-stable reference gene. M < 0.5 indicates high expression stability. Panel (a): Plan A (adopted dataset, most stable pair: *Skip16* + *Ukn2*); Panel (b): Plan B (dataset after removing outlier Ct value for *Ukn2* in root sample).

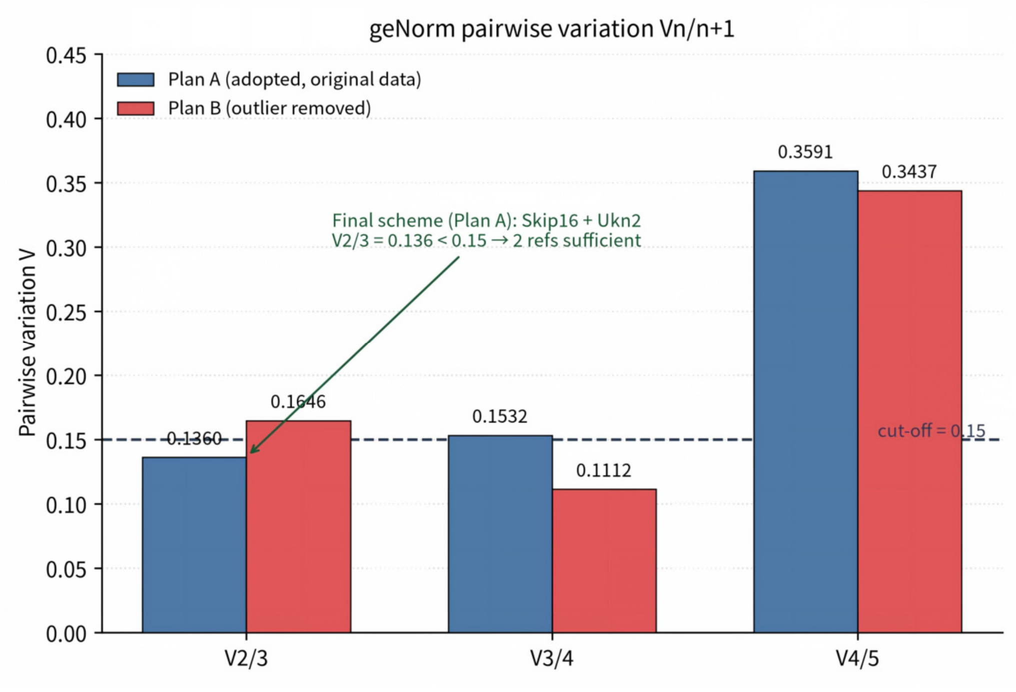

Figure S4. Pairwise variation (Vn/n+1) calculated by geNorm to determine the optimal number of reference genes for normalization. The threshold value 0.15 is marked with red dashed line; V-value below 0.15 means additional reference gene is not required. Blue bars: Plan A dataset; Red bars: Plan B dataset.

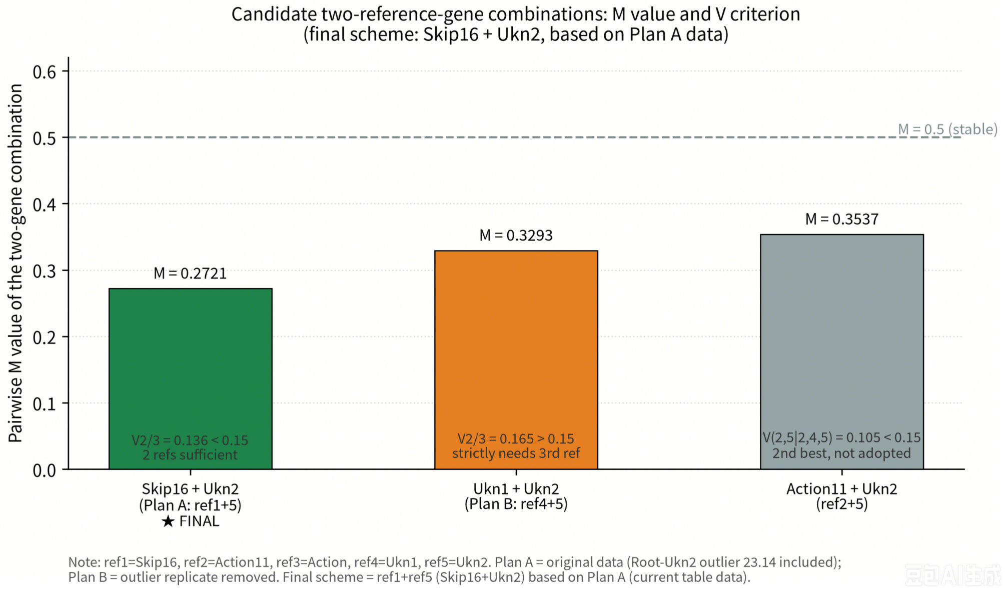

Figure S5. Comparison of pairwise M-values for all two-reference-gene combinations under Plan A dataset. Green bar highlights the finally selected pair *Skip16* + *Ukn2* (★ FINAL). Grey bar shows the second-rank combination *Action11* + *Ukn2* which was not adopted. M = 0.5 threshold is plotted as black dashed line. Gene correspondence: 1-*Skip16*, 2-*Action11*, 3-*Action*, 4-*Ukn1*, 5-*Ukn2*.

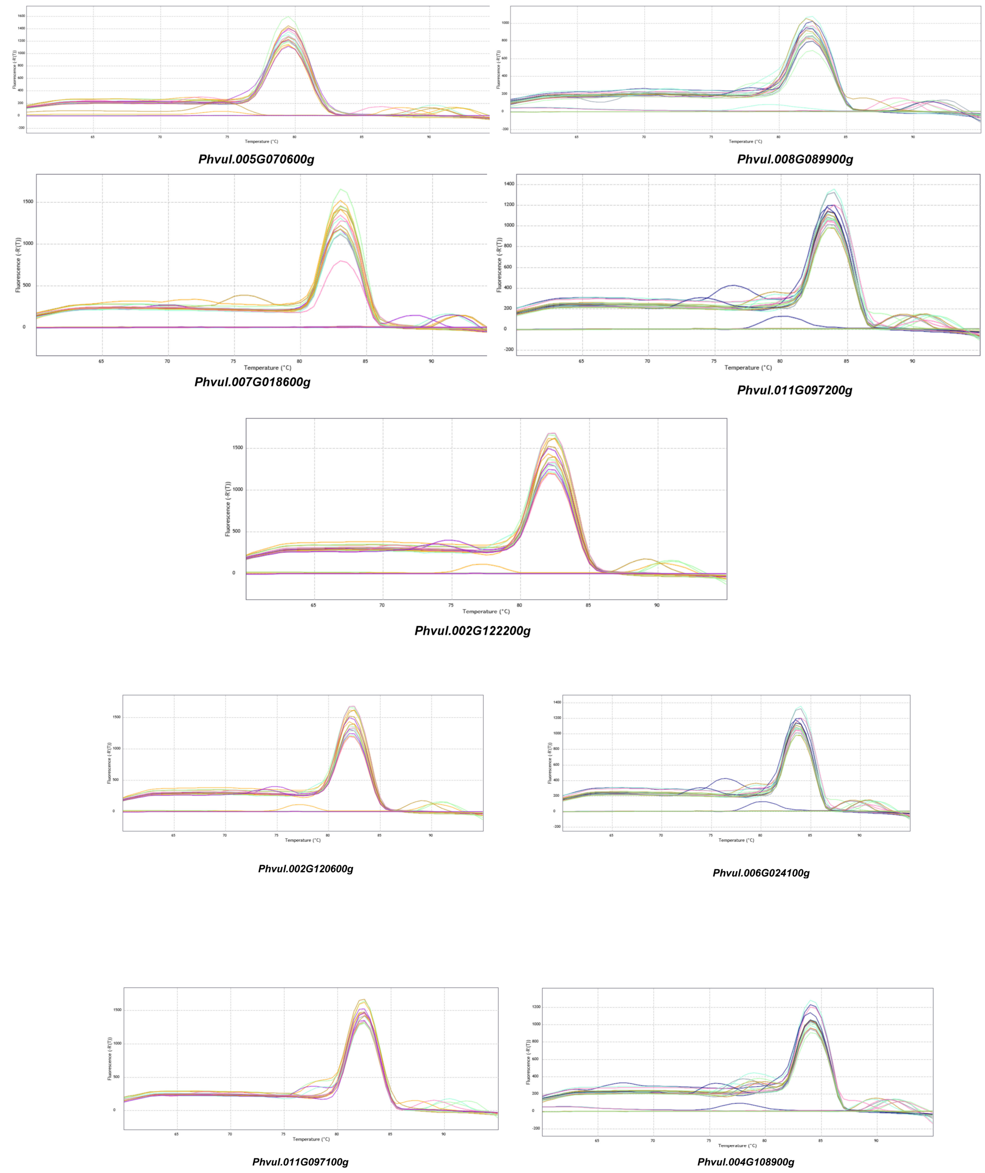

Figure S6. Melting curve analysis of qRT-PCR primers. Melting curves of five target genes and two reference genes (*Skip16*, *Ukn2*) showed single dominant peaks, confirming the specificity of each primer pair. Tm values were consistent across all six biological samples.

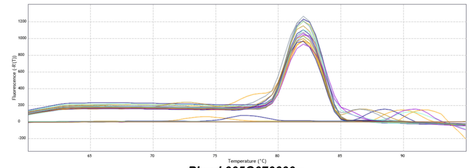

Phvul.005G070600g

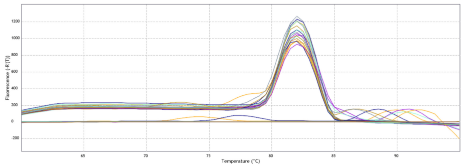

Phvul.008G089900g

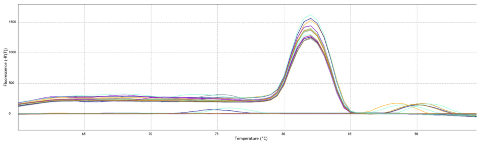

Phvul.007G081600g

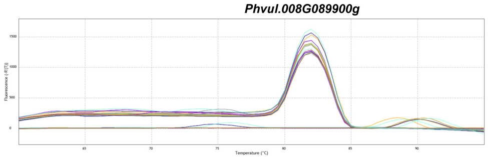

Phvul.011G097200g

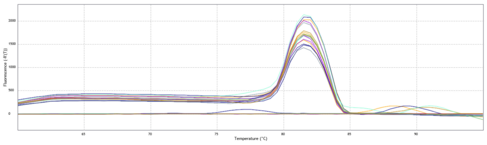

Phvul.002G122200g

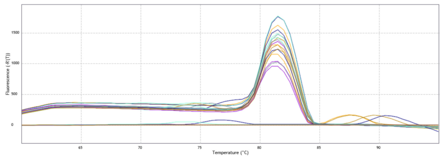

Phvul.002G120600g

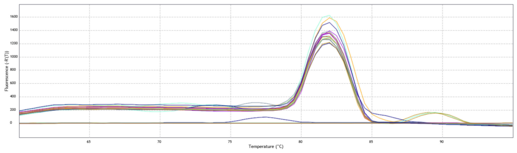

Phvul.006G024100g

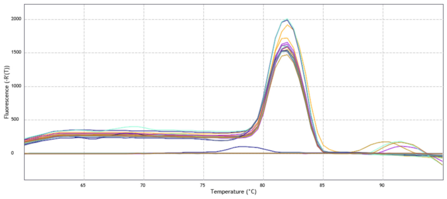

Phvul.011G097100g

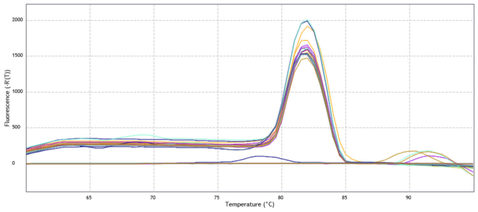

Phvul.004G108900g

Figure S7. Melting curve analysis of candidate reference genes. In all biological replicates, a dominant melting peak (Tm ≈ 82 ° C) was observed for most candidate genes, confirming specific amplification.

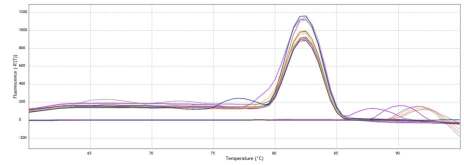

Phvul.005G070600g

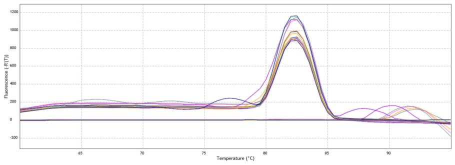

Phvul.008G089900g

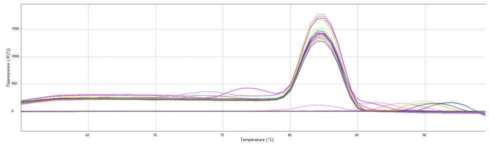

Phvul.007G081600g

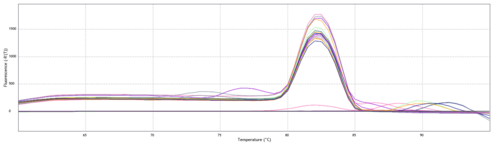

Phvul.011G097200g

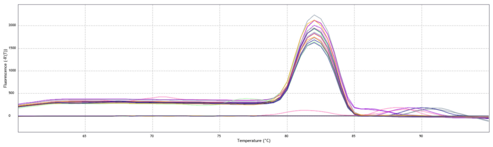

Phvul.002G122200g

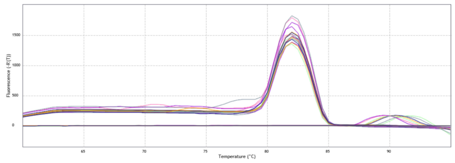

Phvul.002G120600g

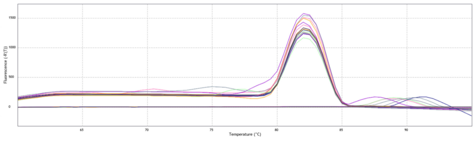

Phvul.006G024100g

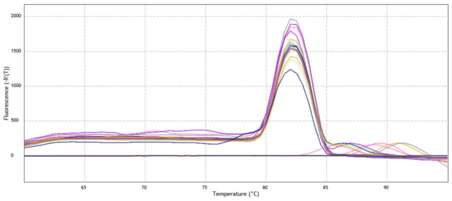

Phvul.011G097100g

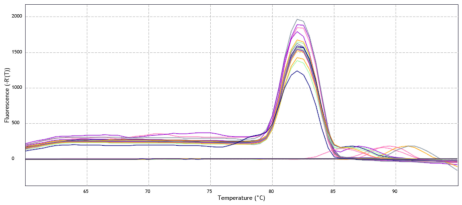

Phvul.004G108900g

Figure S8. Melting curve analysis of candidate reference genes. In all biological replicates, a dominant melting peak (Tm ≈ 82 ° C) was observed for all nine genes, confirming the absence of specific amplification and primer dimer formation.

Table S1. Pairing comparison of photosynthetic pigment content between Jinguan and *pcm*s at different days after flowering (DAF).

Pigment	Time-point (DAF)	Jinguan Mean	*pcm* Mean	Mean difference (J−p)	95 % CI of difference	Adjusted P-value	Significance
Total-chlorophyll	6d	0.4263	0.3333	0.09299	0.04440-0.1416	0.0047	**
	9d	0.08661	0.06808	0.01853	0.01251-0.02454	0.0054	**
	12d	0.02109	0.02412	-0.003025	-0.03060-0.02455	0.9964	ns
	15d	0.01868	0.01573	0.002947	-0.03553-0.04142	0.9913	ns
	18d	0.007051	0.009920	-0.002869	-0.01039-0.004654	0.5783	ns
	21d	0.007100	0.005587	0.001513	-0.01091-0.01394	0.9691	ns
Chlorophyll a	6d	0.2829	0.2229	0.05999	0.04981-0.07017	< 0.0001	****
	9d	0.08661	0.04756	0.03904	0.02886-0.04923	< 0.0001	****
	12d	0.01339	0.01257	0.0008218	−0.009359-0.01100	> 0.9999	ns
	15d	0.009201	0.00884	0.0003611	−0.009820-0.01054	> 0.9999	ns
	18d	0.002259	0.003803	−0.001544	−0.01172-0.008637	0.9987	ns
	21d	0.001911	0.002322	−0.0004109	−0.01059-0.009770	> 0.9999	ns
Chlorophyll b	6d	0.1434	0.1104	0.033	0.01462-0.05137	0.0075	**
	9d	0.08661	0.02052	0.06609	0.06360-0.06858	< 0.0001	****
	12d	0.007706	0.01155	−0.003847	−0.01007-0.002374	0.2076	ns
	15d	0.009476	0.00689	0.002586	−0.005848-0.01102	0.5895	ns
	18d	0.004793	0.006118	−0.001325	−0.007746-0.005096	0.8971	ns
	21d	0.005189	0.003265	0.001924	−0.009585-0.01343	0.8292	ns
Carotenoid	6d	0.146	0.1142	0.0318	0.009120-0.05447	0.0191	*
	9d	0.08661	0.02538	0.06123	0.05715-0.06531	0.0002	***
	12d	0.01487	0.01669	−0.001826	−0.01468-0.01103	0.9884	ns
	15d	0.01148	0.01104	0.000445	−0.009639-0.01053	> 0.9999	ns
	18d	0.00661	0.01262	−0.006009	−0.01815-0.006135	0.1942	ns

	21d	0.006266	0.0131	−0.006837	−0.01143-0.002249	0.0148	*

Note：The average difference is equal to the Jinguan minus *pcm*. There is a 95% confidence interval. *$P < 0.05$, **$P < 0.01$, ***$P < 0.001$, $P < 0.0001$; ns，there is no significant difference.

Table S2. Transcriptome data quality statistics.

Sample	Raw Reads (M)	Raw Base	Clean Reads	Clean Base	Clean Reads Q20 (%)	Clean Reads Q30 (%)	GC content（%）
WT-1	48702730	7.31	42497762	6.37	99.29	97.36	44.18
WT-2	50599606	7.59	45646980	6.85	99.36	97.59	44.18
WT-3	44931422	6.74	45312690	6.8	99.34	97.51	44.08
M-1	43183210	6.48	47685612	7.15	99.29	97.33	44.03
M-2	46368822	6.96	49741190	7.46	99.34	97.55	44.19
M-3	45912636	6.89	44384744	6.66	99.35	97.57	44.01

Table S3. Transcriptome data sequence alignment reference genome.

Sample	Clean Reads	Mapped Reads	Unique Mapped Reads	Multi-Mapped Reads
WT-1	47685612	46303498(97.10%)	45147113(94.68%)	1156385(2.43%)
WT-2	49741190	48256199(97.01%)	46939942(94.37%)	1316257(2.65%)
WT-3	44384744	43172645(97.27%)	42177061(95.03%)	995584(2.24%)
M-1	42497762	41345801(97.29%)	40455516(95.19%)	890285(2.09%)
M-2	45646980	44326221(97.11%)	43231545(94.71%)	1094676(2.40%)
M-3	45312690	44072775(97.26%)	43077082(95.07%)	995693(2.20%)

Table S4. Annotated table of differential transcription factors.

Name	log2FoldChange	TF_Family	Regulated
Phvul.001G042100g	2.189365828	WRKY	up
Phvul.001G072200g	2.241044686	NAC	up
Phvul.002G015100g	-2.170860292	MYB	down
Phvul.002G035100g	2.722698688	AP2/ERF-ERF	up
Phvul.002G209300g	1.306181364	CAMTA	up
Phvul.002G267800g	1.651605926	AP2/ERF-ERF	up
Phvul.004G075500g	1.475370642	NAC	up
Phvul.004G092100g	2.264458895	AP2/ERF-ERF	up
Phvul.004G122000g	-3.558013581	AP2/ERF-ERF	down
Phvul.005G084500g	2.113302305	NAC	up
Phvul.006G181500g	1.617965084	NAC	up
Phvul.008G001000g	1.312808537	NAC	up
Phvul.008G090300g	-1.846836122	WRKY	down
Phvul.008G098900g	1.735583777	AP2/ERF-ERF	up
Phvul.009G078300g	-3.904233889	HSF	down
Phvul.009G123300g	-3.994642334	AP2/ERF-ERF	down
Phvul.009G137900g	3.222418645	AP2/ERF-ERF	up
Phvul.009G187700g	2.705712581	MYB	up
Phvul.009G210100g	1.198612824	DBP	up
Phvul.010G062500g	-2.576511818	WRKY	down
Phvul.010G161100g	2.672484896	bHLH	up
Phvul.005G036900g	2.069972501	WRKY	up
Phvul.001G042200g	1.178756359	bHLH	up
Phvul.003G085000g	1.704613989	WRKY	up
Phvul.011G119500g	-1.049407632	GRAS	down
Phvul.007G174800g	2.014635272	bHLH	up
Phvul.011G024700g	-2.163015953	HSF	down
Phvul.009G235700g	-1.335099224	NAC	down
Phvul.008G246000g	1.425402546	bHLH	up
Phvul.001G214400g	-1.471642104	AP2/ERF-ERF	down
Phvul.003G1408001g	1.445356109	WRKY	up
Phvul.007G079900g	1.614491123	bHLH	up
Phvul.001G215100g	1.155434707	TUB	up
Phvul.007G067500g	-1.496493623	MYB	down
Phvul.001G170800g	2.81964796	HB-HD-ZIP	up
Phvul.003G2848001g	-1.261336669	C2H2	down
Phvul.005G055400g	1.495134269	PLATZ	up
Phvul.003G128800g	-2.506905983	NAC	down
Phvul.001G140100g	1.147274668	B3-ARF	up
Phvul.008G257400g	1.900996793	bZIP	up
Phvul.009G109600g	2.303940002	LOB	up
Phvul.009G182800g	-2.484261298	HSF	down
Phvul.002G221000g	-1.578577448	AP2/ERF-ERF	down
Phvul.006G090800g	1.822027236	Others	up
Phvul.011G109600g	1.446182774	MYB	up
Phvul.002G055700g	1.294033207	C2H2	up
Phvul.008G098300g	-1.713802912	MYB	down
Phvul.009G003800g	1.43995334	AP2/ERF-ERF	up

Table S5. DEGs in the carotenoid metabolic pathway.

Gene ID	KEGG	KEGG pathway	Functional notes	Homologous genes
Phvul.002G122200g	K09843	ko00906	CYP707A	*AtCYP707A1*（*AT2G29090*）、*AtCYP707A2*（*AT2G28200*）
Phvul.005G070600g	K09843	ko00906	ABA 8'-hydroxylase	*AtCYP707A1*（*AT2G29090*）、*AtCYP707A2*（*AT2G28200*）
Phvul.001G216100g	K09841	ko00906	3(or 17)beta-hydroxysteroid dehydrogenase	*At5g50600*
Phvul.002G120600g	K09840	ko00906	Carotenoid-cleaving dioxygenase 4	*AtCCD4*（*AT4G19170*）
Phvul.011G097100g	K09841	ko00906	Oryzanol A synthase	
Phvul.004G108900g	K15746	ko00906	β-carotene 3-hydroxylase 2	*AT5G52570*
Phvul.006G024100g	K02291	ko00906	15-cis-phytoene synthase（PSY）	*AT5G17230*
Phvul.011G097200g	K09841	ko00906	Isopiperitenol dehydrogenase（ISPD）	
Phvul.007G018600g	K09838	ko00906	FAD-dependent urate hydroxylase	
Phvul.008G089900g	K09841	ko00906	Xanthoxin oxidase（ABA2）	

Table S6. Screening of significant differences in metabolite molecule-related parameters between

Jinguan and *pcm*.

Index	P-value	FDR	Fold_Change	Log_2FC	Type
Carotenoid_04	9.66E-07	4.06E-05	0.488880309	-1.032446796	down
Carotenoid_56	1.30E-04	7.81E-04	2.332043861	1.221594923	up
Carotenoid_17	4.03E-04	0.001411935	57.04629377	5.834061254	up
Carotenoid_12	6.44E-04	0.002079743	9.05994543	3.179502361	up
Carotenoid_41	8.88E-05	7.40E-04	10.65101021	3.412918366	up
Carotenoid_28	0.001007616	0.002494946	Inf	Inf	up
Carotenoid_29	1.06E-04	7.40E-04	Inf	Inf	up
Carotenoid_30	3.08E-05	4.50E-04	Inf	Inf	up
Carotenoid_46	4.07E-05	4.50E-04	Inf	Inf	up
Carotenoid_47	1.86E-04	9.78E-04	Inf	Inf	up
Carotenoid_14	0.002016082	0.003024123	Inf	Inf	up
Carotenoid_32	0.001485087	0.002494946	Inf	Inf	up
Carotenoid_36	0.001103278	0.002494946	Inf	Inf	up
Carotenoid_08	3.74E-04	0.001411935	Inf	Inf	up
Carotenoid_52	0.001216043	0.002494946	Inf	Inf	up
Carotenoid_53	0.004454465	0.005345358	Inf	Inf	up
Carotenoid_26	0.001047676	0.002494946	Inf	Inf	up
Carotenoid_16	0.003110739	0.00421455	Inf	Inf	up
Carotenoid_49	0.002576094	0.003730895	Inf	Inf	up
Carotenoid_70	0.002726755	0.003817457	Inf	Inf	up
Carotenoid_11	0.004077339	0.005345358	0	-Inf	down
Carotenoid_19	0.001311457	0.002494946	Inf	Inf	up
Carotenoid_65	0.001159245	0.002494946	Inf	Inf	up
Carotenoid_09	0.001408783	0.002494946	Inf	Inf	up
Carotenoid_15	0.001091773	0.002494946	Inf	Inf	up
Carotenoid_43	0.004242344	0.005345358	Inf	Inf	up
Carotenoid_37	3.85E-04	0.001411935	Inf	Inf	up
Carotenoid_34	0.001553937	0.002510206	Inf	Inf	up
Carotenoid_33	0.001309405	0.002494946	Inf	Inf	up
Carotenoid_20	0.004994789	0.005827253	Inf	Inf	up
Carotenoid_18	3.14E-04	0.001411935	Inf	Inf	up

Note: inf means infinity. Since fold_change=the average value of the experimental group/the average value of the control group, when the average value of the control group is 0, fold_change is infinity, which is represented by inf.

Table S7. DEM KEGG pathway types.

KEGG_map	Description	Rich_factor_meta	P-value_meta	Rich_factor_gene	P-value
ko01110	Biosynthesis of secondary metabolites	3/11	0.75	285/2100	0.0000
ko01100	Metabolic pathways	2/7	0.636363636	413/3521	0.0140
ko00999	Biosynthesis of various plant secondary metabolites	1/1	0.25	24/162	0.0683
ko00906	Carotenoid biosynthesis	3/12	1	10/71	0.2349
ko01240	Biosynthesis of cofactors	1/1	0.25	26/328	0.969167435

Table S8. Targeted metabolome analysis of carotenoids and integrated transcriptome–metabolome correlation analysis.

No.	Compound ID	Compound	Class	RT (min)	Q1 (Da)	Q3 (Da)	Ion mode	Ionization model	Molecular formula	CAS No.	Calibration equation	r	LLOQ (μg/mL)	ULOQ (μg/mL)	Standard source
1	Carotenoid_01	α-carotene	carotenes	5.96	537.5	123.2	positive	[M+H]+	C40H56	7488-99-5	y = 7.62433e5 x - 27235.73525	0.99831	0.01	25	Commercial authentic standard
2	Carotenoid_04	β-carotene	carotenes	6.31	537.6	177.1	positive	[M+H]+	C40H56	7235-40-7	y = 1.17558e6 x - 10560.65030	0.99557	0.0025	25	Commercial authentic standard
3	Carotenoid_08	antheraxanthin dipalmitate	xanthophylls	7.43	1061	805	positive	[M+H]+	C72H115O5	-	y = 2.85847e6 x - 21572.30327	0.9978			MWDB database (literature-derived)
4	Carotenoid_09	lutein caprate	xanthophylls	6.04	705.7	533.5	positive	[M+H-18]+	C50H74O3	-	y = 2.85847e6 x - 21572.30327	0.9978			MWDB database (literature-derived)
5	Carotenoid_11	lutein myristate	xanthophylls	6.86	761.8	533.5	positive	[M+H-18]+	C54H82O3	-	y = 2.85847e6 x - 21572.30327	0.9978			MWDB database (literature-derived)
6	Carotenoid_12	lutein palmitate	xanthophylls	7.00	789.8	533.5	positive	[M+H-18]+	C56H86O3	-	y = 2.85847e6 x - 21572.30327	0.9978			MWDB database (literature-derived)
7	Carotenoid_14	5,6epoxy-luttein dilaurate	xanthophylls	7.22	749.6	549.5	positive	[M+H-200]+	C64H100O5	-	y = 2.85847e6 x - 21572.30327	0.9978			MWDB database (literature-derived)
8	Carotenoid_15	lutein dilaurate	xanthophylls	7.36	733.5	533.3	positive	[M+H-201]+	C64H101O4	-	y = 2.85847e6 x - 21572.30327	0.9978			MWDB database (literature-derived)
9	Carotenoid_16	5,6epoxy-lutein-caprate-palmitate	xanthophylls	7.19	805.4	549.4	positive	[M+H-172]+	C66H104O5	-	y = 2.85847e6 x - 21572.30327	0.9978			MWDB database (literature-derived)
10	Car	lutein	xanth	7.	76	5	po	[M+	C68	-	y =	0.9			MWDB

No.	Compound ID	Compound	Class	RT (min)	Q1 (Da)	Q3 (Da)	Ion mode	Ionization model	Molecular formula	CAS No.	Calibration equation	r	LLOQ (µg/mL)	ULOQ (µg/mL)	Standard source
	otenoid_17	dimyristate	ophylls	69	1.8	33.5	sitive	H-228]+	H108O4		2.85847e6 x - 21572.30327	978			database (literature-derived)
11	Carotenoid_18	lutein dipalmitate	xanthophylls	7.70	789.8	533.5	positive	[M+H-256]+	C72H116O4	-	y = 2.85847e6 x - 21572.30327	0.9978			MWDB database (literature-derived)
12	Carotenoid_19	lutein distearate	xanthophylls	7.96	817.9	533.5	positive	[M+H-284]+	C76H124O4	-	y = 2.85847e6 x - 21572.30327	0.9978			MWDB database (literature-derived)
13	Carotenoid_20	lutein dioleate	xanthophylls	7.33	815.7	533.4	positive	[M+H-282]+	C76H120O4	-	y = 2.85847e6 x - 21572.30327	0.9978			MWDB database (literature-derived)
14	Carotenoid_21	lutein oleate	xanthophylls	6.56	815.7	533.4	positive	[M+H-18]+	C58H88O3	-	y = 2.85847e6 x - 21572.30327	0.9978			MWDB database (literature-derived)
15	Carotenoid_26	rubixanthin palmitate	xanthophylls	7.58	791.7	535.4	positive	[M+H]+	C56H86O2	-	y = 2.85847e6 x - 21572.30327	0.9978			MWDB database (literature-derived)
16	Carotenoid_28	violaxanthin laurate	xanthophylls	5.72	783.7	583.4	positive	[M+H-18]+	C52H80O6	-	y = 2.85847e6 x - 21572.30327	0.9978			MWDB database (literature-derived)
17	Carotenoid_29	violaxanthin myristate	xanthophylls	6.21	811.8	793.7	positive	[M+H]+	C54H82O5	-	y = 2.85847e6 x - 21572.30327	0.9978			MWDB database (literature-derived)
18	Carotenoid_30	violaxanthin palmitate	xanthophylls	6.38	839.8	821.8	positive	[M+H]+	C56H86O5	-	y = 2.85847e6 x - 21572.30327	0.9978			MWDB database (literature-derived)
19	Carotenoid_32	violaxanthin dilaurate	xanthophylls	6.72	966.7	948.8	positive	[M+H]+	C64H101O6	-	y = 2.85847e6 x - 21572.30327	0.9978			MWDB database (literature-derived)

No.	Compound ID	Compound	Class	RT (min)	Q1 (Da)	Q3 (Da)	Ion mode	Ionization model	Molecular formula	CAS No.	Calibration equation	r	LLOQ (µg/mL)	ULOQ (µg/mL)	Standard source
20	Carotenoid_33	violaxanthin-myristate-caprate	xanthophylls	6.84	965.7	947.8	positive	[M+H]+	C64H100O6	-	y = 2.85847e6 x - 21572.30327	0.9978			MWDB database (literature-derived)
21	Carotenoid_34	violaxanthin-myristate-laurate	xanthophylls	6.88	993.8	975.7	positive	[M+H]+	C66H104O6	-	y = 2.85847e6 x - 21572.30327	0.9978			MWDB database (literature-derived)
22	Carotenoid_36	violaxanthin-myristate-palmitate	xanthophylls	7.22	1050	793.8	positive	[M+H]+	C70H112O6	-	y = 2.85847e6 x - 21572.30327	0.9978			MWDB database (literature-derived)
23	Carotenoid_37	violaxanthin dipalmitate	xanthophylls	7.42	1077.9	821.7	positive	[M+H]+	C72H116O6	-	y = 2.85847e6 x - 21572.30327	0.9978			MWDB database (literature-derived)
24	Carotenoid_41	zeaxanthin palmitate	xanthophylls	7.12	807.8	551.5	positive	[M+H]+	C56H86O3	-	y = 2.85847e6 x - 21572.30327	0.9978			MWDB database (literature-derived)
25	Carotenoid_43	zeaxanthin dilaurate	xanthophylls	7.44	933.9	533.2	positive	[M+H]+	C64H100O4	-	y = 2.85847e6 x - 21572.30327	0.9978			MWDB database (literature-derived)
26	Carotenoid_46	zeaxanthin-laurate-palmitate	xanthophylls	7.67	989.9	533.4	positive	[M+H]+	C68H108O4	-	y = 2.85847e6 x - 21572.30327	0.9978			MWDB database (literature-derived)
27	Carotenoid_47	zeaxanthin-myristate-palmitate	xanthophylls	7.86	1018.1	533.6	positive	[M+H]+	C70H112O4	-	y = 2.85847e6 x - 21572.30327	0.9978			MWDB database (literature-derived)
28	Carotenoid_49	zeaxanthin-palmitate-stearate	xanthophylls	8.21	1074.1	789.8	positive	[M+H]+	C74H120O4	-	y = 2.85847e6 x - 21572.30327	0.9978			MWDB database (literature-derived)
29	Carotenoid_52	β-cryptoxanthin myristate	xanthophylls	7.36	763.9	535.5	positive	[M+H]+	C54H82O2	-	y = 2.85847e6 x - 21572.30	0.9978			MWDB database (literature-derived)

No.	Compound ID	Compound	Class	RT (min)	Q1 (Da)	Q3 (Da)	Ion mode	Ionization model	Molecular formula	CAS No.	Calibration equation	r	LLOQ (μg/mL)	ULOQ (μg/mL)	Standard source
30	Carotenoid_53	β-cryptoxanthin palmitate	xanthophylls	7.58	791.9	535.5	positive	[M+H]+	$C_{56}H_{86}O_2$	-	327 y = 2.85847e6 x - 21572.30327	0.9978			MWDB database (literature-derived)
31	Carotenoid_55	antheraxanthin	xanthophylls	2.99	585.5	175.4	positive	[M+H]+	$C_{40}H_{56}O_3$	640-03-9	y = 6.68817e5 x - 13265.54363	0.99379	0.1	25	Commercial authentic standard
32	Carotenoid_56	zeaxanthin	xanthophylls	4.74	569.4	477.5	positive	[M+H]+	$C_{40}H_{56}O_2$	144-68-3	y = 4.88695e5 x - 5329.86609	0.99854	0.05	25	Commercial authentic standard
33	Carotenoid_57	violaxanthin	xanthophylls	1.61	601.4	565.4	positive	[M+H]+	$C_{40}H_{56}O_4$	126-29-4	y = 5.65540e5 x - 22068.52214	0.99737	0.01	25	Commercial authentic standard
34	Carotenoid_58	neoxanthin	xanthophylls	2.01	601.4	565.5	positive	[M+H]+	$C_{40}H_{56}O_4$	14660-91-4	y = 2.85847e6 x - 21572.30327	0.9978	0.025	25	Commercial authentic standard
35	Carotenoid_59	lutein	xanthophylls	4.18	551.5	175.4	positive	[M+H-18]+	$C_{40}H_{56}O_2$	127-40-2	y = 9.97781e5 x - 3063.53131	0.99818	0.005	25	Commercial authentic standard
36	Carotenoid_60	β-cryptoxanthin	xanthophylls	5.59	553.5	177.4	positive	[M+H]+	$C_{40}H_{56}O$	472-70-8	y = 8.55921e5 x - 19281.83933	0.99521	0.01	25	Commercial authentic standard
37	Carotenoid_61	astaxanthin	xanthophylls	3.52	597.3	147.1	positive	[M+H]+	$C_{40}H_{52}O_4$	472-61-7	y = 6.07528e6 x - 16887.13781	0.99536	0.5	25	Commercial authentic standard
38	Carotenoid_64	α-cryptoxanthin	xanthophylls	5.14	553.5	123.1	positive	[M+H]+	$C_{40}H_{56}O$	24480-38-4	y = 1.00913e6 x - 3709.97066	0.99754	0.0005	25	Commercial authentic standard
39	Carotenoid_65	capsorubin	xanthophylls	4.48	601.4	109	positive	[M+H]+	$C_{40}H_{56}O_4$	470-38-2	y = 2.31670e6 x - 2353	0.99604	0.05	25	Commercial authentic standar

No.	Compound ID	Compound	Class	RT (min)	Q1 (Da)	Q3 (Da)	Ion mode	Ionization model	Molecular formula	CAS No.	Calibration equation	r	LLOQ (μg/mL)	ULOQ (μg/mL)	Standard source
											.82234				d
40	Carotenoid_67	echinenone	xanthophylls	5.60	551.6	203.1	positive	[M+H]+	$C_{40}H_{54}O$	432-68-8	y = 2.50391e7 x - 19468.29127	0.99708	0.0005	25	Commercial authentic standard
41	Carotenoid_69	Fucoxanthin	xanthophylls	1.12	659.7	641.6	positive	[M+H]+	$C_{42}H_{58}O_6$	3351-86-8	y = 2.48510e6 x - 1862.02843	0.99659	0.05	25	Commercial authentic standard
42	Carotenoid_70	Adonirubin	xanthophylls	4.38	581.3	203	positive	[M+H]+	$C_{40}H_{52}O_3$	4418-72-8	y = 2.63754e6 x - 5137.73001	0.99502	0.1	25	Commercial authentic standard
1	Carotenoid_01	α-carotene	carotenes	5.96	537.5	123.2	positive	[M+H]+	$C_{40}H_{56}$	7488-99-5	y = 7.62433e5 x - 27235.73525	0.99831	0.01	25	Commercial authentic standard
2	Carotenoid_04	β-carotene	carotenes	6.31	537.6	177.1	positive	[M+H]+	$C_{40}H_{56}$	7235-40-7	y = 1.17558e6 x - 10560.65030	0.99557	0.0025	25	Commercial authentic standard
3	Carotenoid_08	antheraxanthin dipalmitate	xanthophylls	7.43	1061	805	positive	[M+H]+	$C_{72}H_{115}O_5$	-	y = 2.85847e6 x - 21572.30327	0.9978			MWDB database (literature-derived)
4	Carotenoid_09	lutein caprate	xanthophylls	6.04	705.7	533.5	positive	[M+H-18]+	$C_{50}H_{74}O_3$	-	y = 2.85847e6 x - 21572.30327	0.9978			MWDB database (literature-derived)
5	Carotenoid_11	lutein myristate	xanthophylls	6.86	761.8	533.5	positive	[M+H-18]+	$C_{54}H_{82}O_3$	-	y = 2.85847e6 x - 21572.30327	0.9978			MWDB database (literature-derived)
6	Carotenoid_12	lutein palmitate	xanthophylls	7.00	789.8	533.5	positive	[M+H-18]+	$C_{56}H_{86}O_3$	-	y = 2.85847e6 x - 21572.30327	0.9978			MWDB database (literature-derived)
7	Carotenoid_1	5,6epoxy-luttein dilaurate	xanthophylls	7.22	749.6	549.5	positive	[M+H-200]+	$C_{64}H_{100}O_5$	-	y = 2.85847e6 x -	0.9978			MWDB database (literatu

No.	Compound ID	Compound	Class	RT (min)	Q1 (Da)	Q3 (Da)	Ion mode	Ionization model	Molecular formula	CAS No.	Calibration equation	r	LLOQ (μg/mL)	ULOQ (μg/mL)	Standard source
	4										21572.30327				re-derived)
8	Carotenoid_15	lutein dilaurate	xanthophylls	7.36	733.5	533.3	positive	[M+H-201]+	$C_{64}H_{101}O_4$	-	y = 2.85847e6 x - 21572.30327	0.9978			MWDB database (literature-derived)
9	Carotenoid_16	5,6epoxy-lutein-caprate-palmitate	xanthophylls	7.19	805.4	549.4	positive	[M+H-172]+	$C_{66}H_{104}O_5$	-	y = 2.85847e6 x - 21572.30327	0.9978			MWDB database (literature-derived)
10	Carotenoid_17	lutein dimyristate	xanthophylls	7.69	761.8	533.5	positive	[M+H-228]+	$C_{68}H_{108}O_4$	-	y = 2.85847e6 x - 21572.30327	0.9978			MWDB database (literature-derived)
11	Carotenoid_18	lutein dipalmitate	xanthophylls	7.70	789.8	533.5	positive	[M+H-256]+	$C_{72}H_{116}O_4$	-	y = 2.85847e6 x - 21572.30327	0.9978			MWDB database (literature-derived)
12	Carotenoid_19	lutein distearate	xanthophylls	7.96	817.9	533.5	positive	[M+H-284]+	$C_{76}H_{124}O_4$	-	y = 2.85847e6 x - 21572.30327	0.9978			MWDB database (literature-derived)
13	Carotenoid_20	lutein dioleate	xanthophylls	7.33	815.7	533.4	positive	[M+H-282]+	$C_{76}H_{120}O_4$	-	y = 2.85847e6 x - 21572.30327	0.9978			MWDB database (literature-derived)
14	Carotenoid_21	lutein oleate	xanthophylls	6.56	815.7	533.4	positive	[M+H-18]+	$C_{58}H_{88}O_3$	-	y = 2.85847e6 x - 21572.30327	0.9978			MWDB database (literature-derived)
15	Carotenoid_26	rubixanthin palmitate	xanthophylls	7.58	791.7	535.4	positive	[M+H]+	$C_{56}H_{86}O_2$	-	y = 2.85847e6 x - 21572.30327	0.9978			MWDB database (literature-derived)
16	Carotenoid_28	violaxanthin laurate	xanthophylls	5.72	783.7	583.4	positive	[M+H-18]+	$C_{52}H_{80}O_6$	-	y = 2.85847e6 x - 21572.30327	0.9978			MWDB database (literature-derived)
17	Carotenoi	violaxanthin myristate	xanthophylls	6.21	811.8	793.	positive	[M+H]+	$C_{54}H_{82}O_5$	-	y = 2.85847e	0.9978			MWDB database

No.	Compound ID	Compound	Class	RT (min)	Q1 (Da)	Q3 (Da)	Ion mode	Ionization model	Molecular formula	CAS No.	Calibration equation	r	LLOQ (μg/mL)	ULOQ (μg/mL)	Standard source
	d_29					7					6 x - 21572.30327				(literature-derived)
18	Carotenoid_30	violaxanthin palmitate	xanthophylls	6.38	839.8	821.8	positive	[M+H]+	$C_{56}H_{86}O_5$	-	y = 2.85847e6 x - 21572.30327	0.9978			MWDB database (literature-derived)
19	Carotenoid_32	violaxanthin dilaurate	xanthophylls	6.72	966.7	948.8	positive	[M+H]+	$C_{64}H_{101}O_6$	-	y = 2.85847e6 x - 21572.30327	0.9978			MWDB database (literature-derived)
20	Carotenoid_33	violaxanthin-myristate-caprate	xanthophylls	6.84	965.7	947.8	positive	[M+H]+	$C_{64}H_{100}O_6$	-	y = 2.85847e6 x - 21572.30327	0.9978			MWDB database (literature-derived)
21	Carotenoid_34	violaxanthin-myristate-laurate	xanthophylls	6.88	993.8	975.7	positive	[M+H]+	$C_{66}H_{104}O_6$	-	y = 2.85847e6 x - 21572.30327	0.9978			MWDB database (literature-derived)
22	Carotenoid_36	violaxanthin-myristate-palmitate	xanthophylls	7.22	1050	793.8	positive	[M+H]+	$C_{70}H_{112}O_6$	-	y = 2.85847e6 x - 21572.30327	0.9978			MWDB database (literature-derived)
23	Carotenoid_37	violaxanthin dipalmitate	xanthophylls	7.42	1077.9	821.7	positive	[M+H]+	$C_{72}H_{116}O_6$	-	y = 2.85847e6 x - 21572.30327	0.9978			MWDB database (literature-derived)
24	Carotenoid_41	zeaxanthin palmitate	xanthophylls	7.12	807.8	551.5	positive	[M+H]+	$C_{56}H_{86}O_3$	-	y = 2.85847e6 x - 21572.30327	0.9978			MWDB database (literature-derived)
25	Carotenoid_43	zeaxanthin dilaurate	xanthophylls	7.44	933.9	533.2	positive	[M+H]+	$C_{64}H_{100}O_4$	-	y = 2.85847e6 x - 21572.30327	0.9978			MWDB database (literature-derived)
26	Carotenoid_46	zeaxanthin-laurate-palmitate	xanthophylls	7.67	989.9	533.4	positive	[M+H]+	$C_{68}H_{108}O_4$	-	y = 2.85847e6 x - 21572.30327	0.9978			MWDB database (literature-derived)
27	Carote	zeaxanthin-myristate-palm	xanthophyl	7.8	1018	53	positi	[M+H]+	$C_{70}H_{11}$	-	y = 2.85	0.997			MWDB databas

No.	Compound ID	Compound	Class	RT (min)	Q1 (Da)	Q3 (Da)	Ion mode	Ionization model	Molecular formula	CAS No.	Calibration equation	r	LLOQ (μg/mL)	ULOQ (μg/mL)	Standard source
	noid_47	itate	ls	6	.1	3.6	ve		2O4		847e6 x - 21572.30327	8			e (literature-derived)
28	Carotenoid_49	zeaxanthin-palmitate-stearate	xanthophyls	8.21	1074.1	789.8	positive	[M+H]+	$C_{74}H_{120}O_4$	-	y = 2.85847e6 x - 21572.30327	0.9978			MWDB database (literature-derived)
29	Carotenoid_52	β-cryptoxanthin myristate	xanthophyls	7.36	763.9	535.5	positive	[M+H]+	$C_{54}H_{82}O_2$	-	y = 2.85847e6 x - 21572.30327	0.9978			MWDB database (literature-derived)
30	Carotenoid_53	β-cryptoxanthin palmitate	xanthophyls	7.58	791.9	535.5	positive	[M+H]+	$C_{56}H_{86}O_2$	-	y = 2.85847e6 x - 21572.30327	0.9978			MWDB database (literature-derived)
31	Carotenoid_55	antheraxanthin	xanthophyls	2.99	585.5	175.4	positive	[M+H]+	$C_{40}H_{56}O_3$	640-03-9	y = 6.68817e5 x - 13265.54363	0.99379	0.1	25	Commercial authentic standard
32	Carotenoid_56	zeaxanthin	xanthophyls	4.74	569.4	477.5	positive	[M+H]+	$C_{40}H_{56}O_2$	144-68-3	y = 4.88695e5 x - 5329.86609	0.99854	0.05	25	Commercial authentic standard
33	Carotenoid_57	violaxanthin	xanthophyls	1.61	601.4	565.4	positive	[M+H]+	$C_{40}H_{56}O_4$	126-29-4	y = 5.65540e5 x - 22068.52214	0.99737	0.01	25	Commercial authentic standard
34	Car	neoxanthin	xanth	2.	60	5	po	[M+	C40	146	y =	0.9	0.0	25	Comme

No.	Compound ID	Compound	Class	RT (min)	Q1 (Da)	Q3 (Da)	Ion mode	Ionization model	Molecular formula	CAS No.	Calibration equation	r	LLOQ (μg/mL)	ULOQ (μg/mL)	Standard source
	otenoid_58		ophyls	01	1.4	65.5	sitive	H]+	H56O4	60-91-4	2.85847e6 x - 21572.30327	978	25		rcial authentic standard
35	Carotenoid_59	lutein	xanthophylls	4.18	551.5	175.4	positive	[M+H-18]+	C40H56O2	127-40-2	y = 9.97781e5 x - 3063.53131	0.99818	0.005	25	Commercial authentic standard
36	Carotenoid_60	β-cryptoxanthin	xanthophylls	5.59	553.5	177.4	positive	[M+H]+	C40H56O	472-70-8	y = 8.55921e5 x - 19281.83933	0.99521	0.01	25	Commercial authentic standard
37	Carotenoid_61	astaxanthin	xanthophylls	3.52	597.3	147.1	positive	[M+H]+	C40H52O4	472-61-7	y = 6.07528e6 x - 16887.13781	0.99536	0.5	25	Commercial authentic standard
38	Carotenoid_64	α-cryptoxanthin	xanthophylls	5.14	553.5	123.1	positive	[M+H]+	C40H56O	24480-38-4	y = 1.00913e6 x - 3709.97066	0.99754	0.0005	25	Commercial authentic standard
39	Carotenoid_65	capsorubin	xanthophylls	4.48	601.4	109	positive	[M+H]+	C40H56O4	470-38-2	y = 2.31670e6 x - 2353.82234	0.99604	0.005	25	Commercial authentic standard

No.	Compound ID	Compound	Class	RT (min)	Q1 (Da)	Q3 (Da)	Ion mode	Ionization model	Molecular formula	CAS No.	Calibration equation	r	LLOQ (μg/mL)	ULOQ (μg/mL)	Standard source
40	Carotenoid_67	echinenone	xanthophyls	5.60	551.6	203.1	positive	[M+H]+	$C_{40}H_{54}O$	432-68-8	y = 2.50391e7 x - 19468.29127	0.99708	0.0005	25	Commercial authentic standard
41	Carotenoid_69	Fucoxanthin	xanthophyls	1.12	659.7	641.6	positive	[M+H]+	$C_{42}H_{58}O_6$	3351-86-8	y = 2.48510e6 x - 1862.02843	0.99659	0.05	25	Commercial authentic standard
42	Carotenoid_70	Adonirubin	xanthophyls	4.38	581.3	203	positive	[M+H]+	$C_{40}H_{52}O_3$	4418-72-8	y = 2.63754e6 x - 5137.73001	0.99502	0.1	25	Commercial authentic standard

Table S9. Primers used for qRT-PCR.

Gene ID （Phytozomev2.1）	Gene name	Primer sequence (5′-3′)	Homologous genes	GenBank protein accession	Amplicon size (bp)
Phvul.002G122200g	ABA 8′-hydroxylase （CYP707A1）	F:CCATTAACGTTCCGGGGACA<br>R:TTGTTCCTCTGCCTCCTGC	*AtCYP707A1* （*AT2G29090*）<br>*AtCYP707A2* （*AT2G28200*）	XM_068612682.1	104
Phvul.005G070600g	ABA 8'-hydroxylase	F:GTGGACATGGAATTCAGCACC<br>R:CAATGCAACCGAGAGGGAGT	*AtCYP707A1* （*AT2G29090*）<br>*AtCYP707A2* （*AT2G28200*）	XM_068612682.1	127
Phvul.002G120600g	Carotenoid-cleaving dioxygenase 4（CCD4)	F:ACAATCGGCGGTTACGACTT<br>TGCTTGTTTCCCGTTAGGTC	R: *AtCCD4*（*AT4G19170*）	XM_068612661.1	155
Phvul.011G097100g	Oryzanol A synthase	F:GGCCGAAGCTGCTCTTTACT<br>R:CTCAGACCGGCCAACAGAAA		XM_068636446.11	118
Phvul.004G108900g	β-carotene 3-hydroxylase 2(BCH)	F:GAAGAGGTGGGAGGGCTAGA<br>R:ACAACAACACCGCCTTCCTA	*AT5G52570*	XM_068646610.1	135
Phvul.006G024100g	15-cis-phytoene synthase （PSY）	F:GAGCCAGAGGGTTTGGAC<br>R:TTCTGCTCCTTGACCAGTGC	*AT5G17230*	XM_068638584.1	138
Phvul.011G097200g	Isopiperitenol dehydrogenase（ISPD）	F:CATAATCGCAACCGCAAGCA<br>R:GTATCCCAAGGGTTCCAAGC		XM_068621301.1	128
Phvul.007G018600g	FAD-dependent urate hydroxylase	F:AATCAGGGGTTGTGCAGAGG<br>R:CTTTCTCTTGGCTGGTGGGT		XM_068634346.1	152
Phvul.008G089900g	Xanthoxin oxidase （ABA2）	F:GTTCAACAATGCTGGGGTGC<br>R:TTCATCCCCAAAGCCACTCC		XM_068633598.1	120
Skip16		F:CACCAGGATGCAAAAGTGG<br>R:ATCCGCTTGTCCCTTGAAC			
Ukn2		F:CCAATTCAACCATCCCTCAC<br>R:AAACTCCTCTGCACCCTCAG			
